# Financial impact of sheeppox and goatpox and estimated profitability of vaccination for subsistence farmers in selected northern states of Nigeria

**DOI:** 10.1016/j.prevetmed.2021.105503

**Published:** 2022-01

**Authors:** Megan E. Rawlins, Georgina Limon, Adeyinka J. Adedeji, Sandra I. Ijoma, Rebecca B. Atai, Jolly A. Adole, Banenat B. Dogonyaro, Atuman Y. Joel, Philippa M. Beard, Pablo Alarcon

**Affiliations:** a Royal Veterinary College, Hawkshead Lane, Hatfield, AL9 7TA, United Kingdom; b The Pirbright Institute, Ash Road, Pirbright, Woking, GU24 0NF, United Kingdom; c National Veterinary Research Institute, Vom, Plateau State, Nigeria; d The Roslin Institute, The University of Edinburgh, Easter Bush Campus, Midlothian, EH25 9RG, United Kingdom

**Keywords:** Sheeppox and goatpox, Nigeria, Vaccination, Economic impact, Partial budget, Gross margin

## Abstract

Sheeppox and goatpox (SGP) are important transboundary diseases, endemic in Nigeria, causing severe clinical manifestations, impacting production, and resulting in economic losses. Vaccination is an effective control measure against SGP in endemic countries but is not currently implemented in Nigeria. This study aimed to estimate SGP financial impact and assess economic viability of SGP vaccination at the herd and regional level under different scenarios in Northern Nigeria. Integrated stochastic production and economic herd models were developed for transhumance and sedentary herds. Models were run for two disease scenarios (severely and slightly affected) and with and without vaccination, with data parameterisation from literature estimates, field survey and authors’ experience. Herd-level net financial impact of the disease and its vaccination was assessed using gross margin (GM) and partial budget analyses. These were then used to assess regional financial impact of disease and profitability of a 3-year vaccination programme using a cost-benefit analysis. The regional-analysis was performed under 0 %, 50 % and 100 % government subsidy scenarios; as a standalone programme or in combination with other existing vaccination programmes; and for risk-based and non-risk-based intervention.

Median SGP losses per reproductive female were £27 (90 % CI: £31-£22), and £5 (90 % CI: £7-£3), in sedentary, and £30 (90 % CI: £41-21), and £7 (90 % CI: £10-£3), in transhumance herds, for severely and slightly affected scenarios respectively. Selling animals at a reduced price, selling fewer young animals, and reduced value of affected animals remaining in the herd were the greatest contributors to farmer’s SGP costs. SGP-affected herds realised a GM reduction of up to 121 % in sedentary and 138 % in transhumance. Median estimated regional SGP cost exceeded £24 million. Herd-level median benefits of vaccination per reproductive female were £23.76 (90 % CI: £19.28-£28.61), and £4.01 (90 % CI: £2.36-£6.31), in sedentary, and £26.85 (90 % CI: £17.99-£37.02) and £7.45 (90 % CI: £3.47-£15.14) in transhumance herds, in severely and slightly affected scenarios, respectively. Median benefit: cost ratio (BCR) for severely affected herds at 50% subsidies was 6.62 (90% CI: 5.30-8.90) for sedentary, and 5.14 (90% CI: 3.31-13.81) for transhumance herds. The regional SGP vaccination standalone programme BCR: 7–27, regional SGP vaccination with existing vaccination programme BCR: 7–228 and vaccinating high-risk areas BCR: 19–439 were found to be economically viable for all subsidy levels explored. Vaccinating low-risk areas only realised benefits with 100 % of government subsidies.

This study further increases understanding of SGP’s impact within Northern Nigeria and demonstrates vaccination is an economically viable control strategy at the herd-level and also regionally, depending on the strategy and government subsidy levels considered.

## 1 Introduction

Nigeria has one of the largest small ruminant populations estimated at 35.4 and 22.1 million goats and sheep respectively (FAO, 2016). Small ruminants in Nigeria typically exist in either extensive transhumance (pastoral) or sedentary (backyard) systems (The World Bank, 2018). Approximately 70 % of the small ruminant population is found in the Northern region, with total estimates for Bauchi, Kaduna and Plateau states at 8.5 million, 1.82 million, and 3 million respectively (KDSG, 2008; BSMANR, 2017). Within these states, small ruminants are generally kept for subsistence (transhumance or sedentary), rather than commercial purposes. As in other low-middle income countries, small ruminants contribute to people’s livelihoods within Nigeria in a variety of ways, including improving food access and availability, contributing to income through direct sale of animals and by-products, generation of employment opportunities through production and associated value chains, and their use as banks and insurance (Dominguez-Salas et al., 2019). However, subsistence producers within Nigeria face constraints to optimising their production including shocks such as natural disasters and epidemic and endemic diseases (Aphunu and Okoedo-Okojie, 2011).

Sheeppox and goatpox (SGP) are transboundary small ruminant diseases, caused by sheeppox virus and goatpox virus, of the *Capripoxvirus* genus, family *Poxviridae* (Bhanuprakash et al., 2012). SGP is considered endemic in Nigeria (Gelaye and Lamien, 2019). Virus transmission primarily occurs through direct contact or aerosol inhalation (Bhanuprakash et al., 2006). Clinical manifestations include cutaneous lesions, abortion, weight loss and mortality (Bhanuprakash et al., 2011). Morbidity and case-fatality are generally up to 20 % and 40 % respectively, although vary depending on severity and immunological status of the population (Bolajoko et al., 2019; EFSA, 2014; Gambo et al., 2018; Limon et al., 2020).

SGP causes short and long-term economic impacts, with particularly severe impacts on subsistence farmers in low and middle-income countries (Babiuk et al., 2008). Despite acknowledgement of SGP causing substantial hardship, few studies have quantified its economic impact. Recent studies in Northern Nigeria suggest that mortality losses and reduction in value of infected animals are among the most significant contributors to economic loss due to SGP in herds (Bolajoko et al., 2019; Limon et al., 2020). Quantifying SGP economic losses and the potential benefits of an intervention are critical for informed decision-making regarding disease control, enabling evaluation of whether control costs exceed disease losses. One such disease control intervention is vaccination. Vaccination is an effective control measure for SGP in endemic countries, with successes observed in Morocco and Tunisia following 75−80 % vaccination coverage with a live attenuated SGP vaccine (Ben Chehida et al., 2018). However, no official vaccination control programme exists in Nigeria, and, to the authors’ knowledge, no studies have explored the potential economic benefits of vaccination at the herd and regional level in the country. Analysing vaccination economic efficiency and estimating SGP costs using integrated production and economic models will help inform policy and disease control resource allocation.

The aims of this study were to estimate herd and regional-level financial impact of SGP and assess the economic viability of SGP vaccination in Nigeria, to contribute to policy discussions surrounding disease control. We also aimed to develop integrated herd production and economic models which could utilised in future studies on small ruminant diseases within the region and other similar settings.

## 2 Materials and methods

### 2.1 Theoretical considerations and study overview

The study focuses on the knowledge that livestock diseases have an impact on production efficiency that prevent farmers to optimise production and under-utilise their resources or technology (Knight-Jones and Rushton, 2013). Production economic efficiency is often measured through gross margin analysis, and a significant reduction of these will also have an impact on the profitability of farmers. This in turn will impact the capacity to invest and sustain farmers’ livelihoods (Alarcon et al., 2014). Assessment of the financial impact of diseases are used to estimate benefits from vaccination (Nathues et al., 2018). It is important to note that non-financial impact of diseases, such as on cultural practices (animals used as dowries or reputation) or public health impact (due to zoonotic diseases or food security) will also represent an important loss.

Furthermore, decision-making process towards implementation of vaccination requires an analysis of the economic viability of the vaccine and their potential role in protecting international trade and livelihoods (McLeod and Rushton, 2007). In terms of viability, SGP vaccines have been proven effective in several studies, but currently these are absent in Nigeria despite the continuous outbreaks reported in the country. Decision-making in this context therefore requires considerations of cost-sharing programs between public and private sectors and understanding of barriers for such programs.

In this study, we investigate the impact that SGP has on production economic efficiency of transhumance and sedentary herds, as well as calculating the total financial impact of the disease and its vaccination. The study focuses mainly on direct costs caused by SGP, providing a review and conceptual economic framework for these costs. Furthermore, we assess the viability of cost-sharing vaccination programs by providing assessment of the cost of vaccine delivery, which is a critical factor for success and justification of such programs. Although, the benefits of programs can go well beyond farmers’ gains, as it can create externalities benefiting consumers and other stakeholders (Tisdell, 2020), these are not included due to lack of data and uncertainty.

**Fig. 1:**
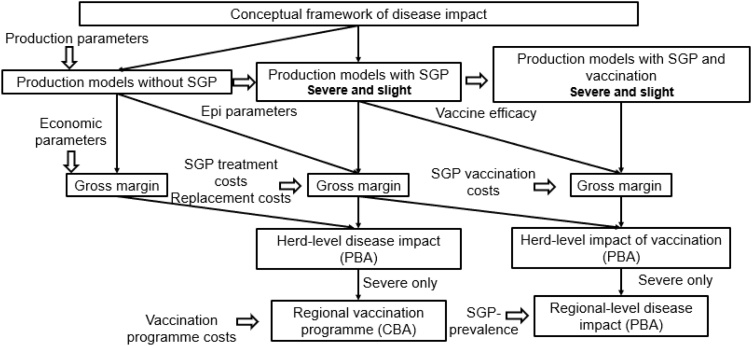
Methodology utilised to develop herd production and farm and regional-level economic models.

The approach used is summarized in Fig. 1.

Analysis comprised of seven stages (1) Developing herd production models without SGP (baseline) and with SGP for slightly and severely affected herds, (2) calculating herd level gross margin (GM) from both models, (3) using partial budget analysis (PBA) to estimate the net SGP cost at the herd and (4) regional level, (5) assess economic viability of vaccination at the herd level, (6) cost-benefit analysis (CBA) to assess economic viability of a 3-year vaccination program at regional level, and (7) conducting sensitivity analyses. The developed herd production and economic models are accessible upon request to the corresponding author.

### 2.2 Study area

Nigeria is located in West Africa and is divided in 36 States, which are further divided into local governments areas (LGAs). This study was conducted in Bauchi, Kaduna, and Plateau States which are located in Northern Nigeria and have 20, 23 and 17 LGA’s respectively. A large proportion of their populations are subsistence farmers (KDSG, 2008). These states were selected as SGP has been demonstrated to cause negative impacts on farmers in this region (Adedeji et al., 2019; Bolajoko et al., 2019; Limon et al., 2020), they encompass the largest small ruminant population in Northern Nigeria, and previous research on small ruminant livestock diseases found the greatest losses in the Northern Savannah region, where Kaduna, Plateau and part of Bauchi are located (Fadiga et al., 2013)

### 2.3 Source of data

Four sources of data were used to parameterise models. In the first instance, a literature review was conducted to produce a conceptual model showing the impact of SGP and to obtain data on herd production parameters such as mortality rates, fecundity, and offtake rates. Focus group discussion (n = 1) with small ruminant farmers (n = 20, 19 sedentary farmers and 1 transhumance farmer), were conducted by authors to obtain some economic parameters not available in the literature. Participants were identified via convenience sampling. Primary data from a survey conducted in 2019 involving 300 randomly selected sedentary herds in Bauchi, Kaduna and Plateau, 100 in each state, was used to obtain herd demographic data and SGP prevalence from the number of households reporting SGP clinical signs (Adedeji et al., 2021). Finally, values missing after literature review, focus group discussion and the survey were obtained from authors who have field and laboratory knowledge and experience in virology, vaccinology, SGP epidemiology, small ruminant husbandry, and animal health programmes within Northern Nigeria.

### 2.4 Herd production models

Transhumance (pastoral) and sedentary (backyard) small ruminant systems are present in Northern Nigeria. They are both extensive husbandry systems, with transhumance systems rearing ruminants alongside low levels of crop-cultivation, with a permanent home-base but seasonal herd movement driven by climatic conditions (Lawal-Adebowale, 2012). In contrast, sedentary systems use local communal grazing and house animals overnight. A more detailed description of these husbandry systems is located in the Supplementary Information. Based on their field experiences, authors estimated that 50–65 % of herds within the study region are transhumance, and 35–50 % sedentary. Due to the differing management practices and disease reactions in transhumance and sedentary herds, SGP costs and vaccination viability need to be estimated for both systems separately, so separate herd production models were developed.

**Fig. 2:**
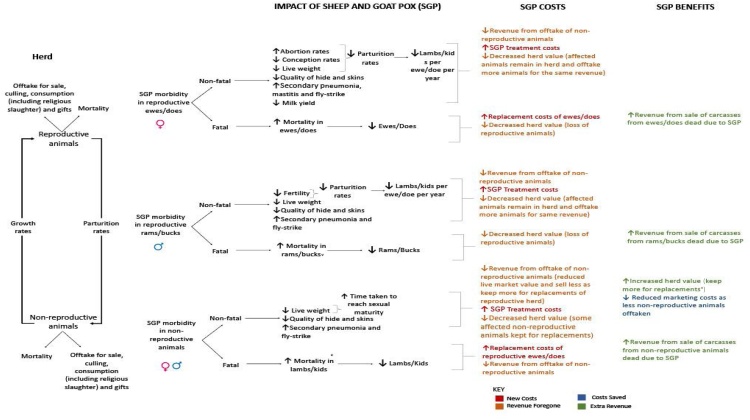
Conceptual framework used in model development depicting the herd production process (left), impacts of SGP on different herd sub-populations (middle), and subsequent effect of SGP impacts on costs and benefits (right). This conceptual framework only includes costs and benefits included within the partial budget model, it is acknowledged that it is not an extensive list of all potential costs and benefits which may arise due to SGP infection.

Herd production models were created in Microsoft Excel, simulating herd population dynamics over an annual production cycle. Basic herd demographic data and production parameters utilised in these production models are presented in Table 1, and further values are found in the Supplementary Information. A conceptual framework was developed as the basis for the herd model and the estimation of disease impact (Fig. 2). A detailed description of the assumptions used in these models and their justification are presented in Table 2.

**Table 1:** Median values for herd demographics and main production parameters utilised for sedentary and transhumance herds in herd production modelling.

	Sedentary Herds	Transhumance Herds
**Sheep**		
Herd size	7	38
Offtake rate	23.80 %	26.30 % - ewes
		30.50 % - rams
Mortality rate (reproductive)	7.50 %	16.10 %
Parturition rate	1.24	1.09
Mortality rate (young)	26.60 %	29.70 %
**Goats**		
Herd size	12	23
Offtake rate	22.85 %	30.20 %
Mortality rate (reproductive)	15.00 % - does	15.00 % - does
	14.49 % - bucks	14.40 % - bucks
Parturition rate	1.68	1.68
Mortality rate (young)	31.70 %	31.70 %

**Table 2:** Assumptions made during modelling, to reflect common practices of farmers within Bauchi, Plateau and Kaduna states. Unless stated otherwise these assumptions apply to both transhumance and sedentary herds.

Assumption	Justification/Reference
***General Herd Management***	
Reproductive herd size remains stable (have the same number of reproductive animals at the beginning and end of the year), unless mortality or other shocks results in deficit of young (non-reproductive) animals for replacement.	Farmers aim to keep herd in equilibrium. Based on authors field experience.
In herds without disease, replacement of the reproductive herd is achieved through rearing homebred animals at no additional costs.	Farmers will avoid expenditure on replacement animals, so replace using own animals if possible. Based on authors field experience.
All young animals are offtaken (i.e. sold), apart from those required for replacement of reproductive herd to keep the herd size stable.	Farmers aim to maximise revenue, while maintaining herd stability. Based on authors field experience.
There are no water or feeding costs in either system.	Transhumance herds use communal water sources, and regularly move to access free grazing. Sedentary herds use streams and wells at no cost, and communal free grazing, which may be supplemented by food waste or excess forage at no cost. Based on authors field experience.
5 % of animals that die, (due to any cause), will be sold as a carcass for marketing at 10 %–20 % of live market value.	Nigerian legislation states that dead animal carcasses should not enter the food chain (Onyimoni et al., 2013). However, authors estimated that 5 % of all animal carcasses are sold into the food chain, based on their field experience.
***SGP and farmers reaction to disease***	
Sheep and goats are simultaneously affected with SGP.	Strains in Nigeria are observed to be equally pathogenic in both species (Bhanuprakash et al., 2012). Additionally, research in Northern Nigeria suggested that both sheep and goats are commonly affected simultaneously (Bolajoko et al., 2019; Limon et al., 2020).
It is not common practice to commercialise sheep and goats milk, and home consumption is low, therefore impact from milk yield reduction due to SGP is considered as negligble.	Research undertaken in Bauchi state found that whilst milk from cattle is commonly sold or consumed in the home, this is not a common practice for sheep and goats milk (Limon et al., 2020).
The increase in veterinary and drug costs as a result of increased secondary pneumonia, mastitis and fly-strike due to disease is negligible so is not considered.	SGP affected animals are commonly offtaken. For those remaining, some farmers will treat for secondary pneumonia, mastitis and fly-strike, at a negligible cost. Treatment costs previously estimated within the study areas and used within the model did not consider veterinary drug costs increase as a result of secondary pneumona, mastitis and fly-strike (Bolajoko et al., 2019; Limon et al., 2020).
Price reduction of live affected animals and affected carcasses is assumed to be homogenous, regardless of reproductive status or sex.	Findings from the study area did not state whether a difference exists with regards to price reduction in affected animals (Bolajoko et al., 2019; Limon et al., 2020).
Farmers will prefer to sell clinically affected animals, instead of providing treatment. 100 % of animals that are clinically affected with SGP and are not offtaken in response to disease, so remain in the herd, will receive treatment to prevent secondary infections and treat pyrexia.	Farmers aim to avoid expenditure for treating clinically affected animals in which the disease may later become fatal, so will offtake animals first, at a lower price than if they were healthy, to avoid treatment expense. Antibiotics and non-steroidal anti-inflammatories are common treatments used. Based on authors field experience.
A proportion of young animals affected with SGP will die or be sold. SGP-affected young animals that remain after death and offtake in response to an SGP outbreak will preferentially be offtaken (at a lower price) to maintain stable numbers of the reproductive herd, rather than selling healthy young animals. These SGP-affected young animals will be sold at a lower price.	Farmers aim to reduce the numbers of affected SGP animals remaining in the herd. Some affected young animals may remain in the herd to allow for adequate replacements to maintain a reproductive herd stability. Based on authors field experience.
Farmers will purchase replacement female reproductive animals (ewes and does) offtaken or dead due to SGP, if they are not able to replace them using own bred young animals. They will purchase the amount required to maintain female reproductive herd equilbrium, up to a maximum of 50 % replacement of female animals offtaken or dead due to SGP.	In the first instance, they will try and replace female animals that have left the herd due to SGP using own bred animals, at no replacement cost. If this is not possible then animals are purchased from other sources e.g. from the market or a middle-man. A maximum of 50 % replacement is based upon knowledge of farmers buying practices and common practice in similar systems. Based on authors field experience.
There is no increase in veterinary service use in SGP outbreak.	Farmers will generally purchase treatment and self-medicate, without seeking veterinary advice or intervention. Based on authors field experience.
***Vaccination***	
100 % of all eligible reproductive and young animals within a herd will be vaccinated.	Eligible animals for SGP vaccination are those greater than 3 months old. Maternally dervied antibody provides immunity until this age (Babiuk et al., 2008). Based on data from the survey in the study area, 97.5 % of young animals were older than 3 months at time of survey. Therefore, 100 % of the reproductive herd and 97.5 % of the young herd are eligible so will be vaccinated (Adedeji et al., 2021)

#### 2.4.1 Herd model without SGP

Initially, herds were modelled without SGP to create baseline models. For this model, the average herd size for sedentary herds was taken from the survey, as previously described, and for transhumance herds from authors estimates based on field experience. The annual production cycle was modelled to begin after lambing/kidding. Four sub-populations were considered within these models: male and female reproductive and young (non-reproductive) animals’ populations. Any animal greater than 1-year old was considered to be reproductive, thus, classifying all young animals remaining at the end of the production cycle as reproductive. This assumption is based upon systematic review data finding small ruminants age of first parturition ranged between 15.9–16.9 months (Otte and Chilonda, 2002), so age at first conception is just over 1 year old. Number of animals within each category were estimated based on authors estimates of the proportions of the herd which are male and female.

For calculations, the same calculations were undertaken for sheep and goat populations and for transhumance and sedentary herds, with different parameter values utilised. R denotes reproductive animals, Y denotes young animals, f denotes female animals, m denotes male animals, 0 denotes beginning of production cycle, 1 denotes end of production cycle and i denotes all populations.

Number of animals which are born ${(N}_{Y,Born})$ was calculated as follows:

$$N_{Y,Born}=\;N_{f,0}\;\times Parturition\;\times Prolificacy$$

Where Parturition is parturition rate; the average number of parturitions per reproductive female per year, and Prolificacy is prolificacy rate; the average number of offspring born per parturition, obtained from the literature. We assumed 50 % were born female and 50 % male.

Number of animals which die during an annual production cycle without SGP ($N_{i-deaths})$ was calculated as follows:

$$N_{i-deaths}=\left(N_{R,0}\;\times {Mortality}_{R}\right)+(N_{Y,0}\;\times {Mortality}_{Y})$$

Where Mortality is the mortality rate in herds without SGP; the proportion of animals which will die over a production cycle, under normal circumstances, obtained from the literature.

Number of reproductive animals offtaken ($N_{R,offtake})\;$ was calculated as follows:

$$N_{R,offtake\;}=N_{R,0}{\;\times {Offtake}_{R}}_{\;}$$

Where Offtake is the offtake rate; the proportion of animals which are sold over an annual production cycle, obtained from the literature.

The number of young animals offtaken in herds is dependent on the number of replacements required to keep the reproductive herd a stable size (replacement rate). Of the young animals remaining after mortalities, all will be offtaken apart from those kept for replacements. Any young animals not offtaken or dead are used as homebred reproductive herd replacements.

Number of replacement females and males required to keep the reproductive herd stable $\left(N_{R,f,replacements\;\;}and\;N_{R,m,replacements\;\;}\right)$ were calculated as follows:

$$N_{R,f,replacements\;\;}=N_{R,f,0}-\;N_{R,f,deaths}\;{-\;N}_{R,f,offtake}\;$$

$$N_{R,m,replacements\;\;}=N_{R,m,0}-\;N_{R,m,deaths}\;{-\;N}_{R,m,offtake}$$

Reproductive herd size at the end of the annual production cycle is therefore the number of reproductive animals at the start, minus the number of reproductive animals which have died and been offtaken, plus the number of young animals kept as replacements.

### 2.4.2 Herd model with SGP and unvaccinated

Production models were created for unvaccinated herds with SGP, considering two theoretical scenarios: (1) An SGP severely affected herd, and (2) An SGP slightly affected herd. These two scenarios accounted for variability in morbidity and mortality. Previous studies in Northern Nigeria represent severely affected herds, given study designs focused on farmers who had experienced outbreaks (Bolajoko et al., 2019; Limon et al., 2020). Slightly affected scenarios reflect those previously exposed herds which is assumed have developed some immunity, and hence may experience reduced signs of disease (this scenario is currently theoretical). SGP infection was modelled to occur at the beginning of the production cycle. Epidemiological SGP parameters and their effect were identified in Fig. 2 and these were incorporated to the baseline models in order to simulate disease impact on population dynamics (Table 3). The four key parameters incorporated into the SGP model were morbidity rate, case-fatality rate, abortion rate and proportion of SGP-affected animals offtaken.

**Table 3:** Epidemiological and vaccination parameters utilised for SGP severely affected herds (severe) and SGP slightly affected herds (slight) for sedentary (SE) and transhumance (TH) models and their distributions for stochastic simulations.

Parameter	Sub-population	Model values (range)				Justification/Reference	Distributions used for stochastic simulations
		SE – severe	SE – slight	TH- severe	TH- slight		
Morbidity rate of SGP	Reproductive sheep	0.259 (0.2−0.335)^b^	0.102 (0.045−0.25)^b^	0.259 (0.2−0.335)^a^	0.102 (0.045−0.25)^b^	Morbidity rate for severely affected herds taken from empirical data from field research in Bauchi and Plateau state (Bolajoko et al., 2019; Limon et al., 2020). Morbidity rate for slightly affected herds taken from research in Sudan in North-East Africa, where SGP is endemic and small ruminants exist in extensive systems similar to Nigeria (Ali, 2008)	SE-severe: Uniform
							SE-slight: Uniform
							TH-severe: PERT
							TH-slight: Uniform
	Reproductive goats	0.485 (0.47−0.5)^a^	0.102 (0.045−0.25)^b^	0.566 (0.44−0.608)^a^	0.102 (0.045−0.25)^b^		SE-severe: PERT
							SE-slight: Uniform
							TH-severe: PERT
							TH-slight: Uniform
	Young sheep	0.615 (0.471−0.706)^a^	0.102 (0.045−0.25)^b^	0.503 (0.435−0.642)^a^	0.102 (0.045−0.25)^b^		SE-severe: PERT
							SE-slight: Uniform
							TH-severe: PERT
							TH-slight: Uniform
	Young goats	0.60 (0.47−0.667)^a^	0.102 (0.045−0.25)^b^	0.714 (0.513−0.775)^a^	0.102 (0.045−0.25)^b^		SE-severe: PERT
							SE-slight: Uniform
							TH-severe: PERT
							TH-slight: Uniform
Case-fatality rate of SGP	Reproductive sheep	0.165 (0−0.33)^a^	0.075 (0.5−0.1)^b^	0.24 (0.125−0.893)^a^	0.075 (0.5−0.1)^b^	Case-fatality rate for severely affected herds taken from empirical data from field research in Bauchi and Plateau state (Bolajoko et al., 2019; Limon et al., 2020). Case-fatality rate for slightly affected herds taken from literature focusing on various endemic areas (OIE, 2013; USDA, 2016)	SE-severe: Uniform
							SE-slight: Uniform
							TH-severe: PERT
							TH-slight: Uniform
	Reproductive goats	0.65 (0−0.33)^b^	0.075 (0.5−0.1)^b^	0.211 (0.0−0.225)^a^	0.075 (0.5−0.1)^b^		SE-severe: Uniform
							SE-slight: Uniform
							TH-severe: PERT
							TH-slight: Uniform
	Young sheep	0.5 (0.367−0.691)^a^	0.075 (0.5−0.1)^b^	0.503 (0.435−0.642)^a^	0.075 (0.5−0.1)^b^		SE-severe: PERT
							SE-slight: Uniform
							TH-severe: PERT
							TH-slight: Uniform
	Young goats	0.5 (0.286−0.667)^a^	0.075 (0.5−0.1) b	0.476 (0.319−0.617)^a^	0.075 (0.5−0.1)^b^		SE-severe: PERT
							SE-slight: Uniform
							TH-severe: PERT
							TH-slight: Uniform
Proportion of SGP-affected reproductive and young animals offtaken	Sheep	0.314 (0−0.4)^a^	0.314 (0−0.4)^a^	0.376 (0.052−0.833)^a^	0.376 (0.052−0.833)^a^	Farmers will offtake a proportion of SGP-affected animals at a lower price, in response to an outbreak. This aims to generate revenue and rather than keeping animals which later die of SGP, and to prevent further disease spread in the herd. Offtake rates of SGP-affected animals in severely affected herds were obtained from previous research in Bauchi state (Limon et al., 2020). It was assumed this parameter is the same in severely affected herds.	SE-severe: PERT
							SE-slight: PERT
							TH-severe: PERT
							TH-slight: PERT
	Goats	0.374 (0.2−0.5)^a^	0.374 (0.2−0.5)^a^	0.386 (0.203−0.489)^a^	0.386 (0.203−0.489)^a^		SE-severe: PERT
							SE-slight: PERT
							TH-severe: PERT
							TH-slight: PERT
Abortion rate of SGP-affected animals	Reproductive sheep and goats	0.03	0.03	0.03	0.03	Some reproductive females affected with SGP will abort (EFSA, 2014). No distinction is made in the literature with regards to differing abortion rates dependent on outbreak severity, so values are assumed to be the same for both scenarios.	Non-distributed
Efficacy of SGP vaccination	All animals	0.9 (0.8−1)^b^	0.9 (0.8−1)^b^	0.9 (0.8−1)^b^	0.9 (0.8−1)^b^	Efficacy values of RM65 and Gorgan vaccination strains, (the most common vaccination strains used), from vaccination challenge studies were used in the model. An SGP vaccination has been developed in Nigeria, but its efficacy has not yet been evaluated (Abbas et al., 2010; EFSA, 2014).	SE-severe: Uniform
							SE-slight: Uniform
							TH-severe: Uniform
							TH-slight: Uniform

a Range is the 1st and 3rd quartile, median used in the deterministic model.

b Range is the lowest and highest value, mean used in the deterministic model.

For calculating the number of young animals born in herds with SGP$\;{(N}_{Y,born,SGP})$, SGP abortion rate was incorporated, as follows:

$$N_{Y,born,SGP}=N_{f,0}\;\times Parturition\;\times \left(1-Abortion\right)\times Prolificacy\;$$

Where Abortion is the abortion rate; the proportion of reproductive females clinically affected with SGP which abort, obtained from the literature.

Numbers of SGP-affected animals were calculated using SGP morbidity rate; the proportion of animals that become clinically affected with SGP. Total number of SGP-affected animals ${(N}_{i,SGP})$ was calculated as follows:

$$N_{i,SGP}={(N}_{R,0}{\;\times {Morb}_{R,SGP})}_{\;}+\;{(N}_{Y,0}{\;\times {Morb}_{Y,SGP})}_{\;}$$

Where Morb is the SGP morbidity-rate, obtained for severely affected scenarios from research in the study area, and for slightly affected scenarios from endemic countries where small ruminants exist in similar extensive systems.

SGP case-fatality rates; the proportion of animals clinically affected with SGP that die due to the disease, were used to calculate the additional deaths in the herd due to SGP ${(N}_{i,SGP,deaths})$. Total number of animals which die due to SGP was calculated as follows:

$$N_{i,SGP,deaths}=\;{N_{i-SGP\;}\times \;{CFR}_{i-SGP}}_{\;}$$

Where CFR is the case-fatality rate of SGP, obtained for severely affected scenarios from research in the study area, and for slightly affected scenarios from endemic countries where small ruminants exist in similar extensive systems.

Some animals affected with SGP are offtaken as a disease coping mechanism. Total number of SGP-affected animals offtaken ${(N}_{i,SGP,offtake})$ is calculated as follows:

$$N_{i,SGP,offtake}=\;{N_{i-SGP\;}\times \;{Offtake}_{i-SGP}}_{\;}$$

Where Offtake is SGP-affected offtake rate, the proportion of SGP-affected animals which are offtaken, obtained from research in the study area and assumed to be the same in slightly and severely affected scenarios.

Number of healthy (not SGP-affected) reproductive animals offtaken ${(N}_{R,offtake-healthy})$ was calculated as follows:

$$N_{R,offtake-healthy\;}=\frac{\left(N_{R,offtake,baseline\;}\;\times P_{R,healthy}\right)-\left(N_{R,SGP,offtake\;}\times P_{R,SGP}\right)}{P_{R,healthy}}$$

This is based on the assumption that in herds with SGP, after offtaking SGP-affected reproductive animals, farmers will then offtake enough healthy (not affected with SGP) reproductive animals to make the same revenue as in a disease-free scenario. P represents the price i.e. sale value of the animals.

For young animals, the number of healthy (non-SGP affected) animals offtaken depends on the replacements required for reproductive herd stability. It is assumed that farmers will preferentially offtake SGP-affected young animals remaining in the herd following mortality and offtake as a disease coping mechanism, so that they can try to keep as many healthy young as possible for replacements. If the number of SGP-affected animals remaining following death and initial offtake is less than the number of young required for replacements, then all of the SGP-affected animals will be offtaken, with the remainder of the offtake comprising of healthy animals. If the number of SGP-affected animals remaining following death and initial offtake is greater than the number of young required for replacements, then all SGP-affected animals apart from those required for replacements will be offtaken. No healthy animals will be offtaken. In this scenario, some SGP-affected young animals are used as reproductive replacements.

In some of the SGP scenarios modelled, there are not enough young to replace all of the reproductive herd and keep it in equilibrium. In these cases, breeding females are purchased into the herd. It is assumed that farmers will purchase the amount required to maintain female reproductive herd equilibrium, up to a maximum of 50 % replacement of female animals offtaken or dead due to SGP.

Reproductive herd size at the end of the annual production cycle in herds with SGP is therefore the number of reproductive animals at the start, minus the number of reproductive animals which have died due to SGP and those which have died due to other causes, and been reproductive offtake due to SGP or other reasons, plus the number of young animals kept as replacements and the number of purchased replacement breeding females.

#### 2.4.3 Herd model with SGP and vaccinated

Finally, production models were created for vaccinated herds with SGP, considering the SGP severely and slightly affected scenario. Vaccination effects were incorporated into the model to simulate its impact on population dynamics. Vaccine efficacy utilised is presented in Table 3.

Vaccine efficacy: the proportion of animals vaccinated that will not develop clinical disease, under optimal conditions, was utilised to calculate numbers of SGP clinically affected animals. Number of reproductive animals affected with SGP in vaccinated herds $\left(N_{R,SGP}\right)$, was calculated as follows:

$$N_{R,SGP}=N_{R,0}\times \left(1-VE\right)\times \;{Morb}_{R,SGP}\;$$

Where VE is vaccine efficacy. It is assumed that vaccination will be done once per year, and 100 % of animals which are eligible for vaccination (exceeding 3 months of age), will be vaccinated. All of the reproductive herd will therefore be vaccinated.

Number of young animals affected with SGP in vaccinated herds ${\;(N}_{Y-SGP})$ was calculated as follows:

$$N_{Y,SGP}=[\left(N_{Y,Vaccinated\;}\times \left(1-VE\right)\times {Morb}_{Y,SGP}\right)]+[\left(N_{Y,Born,SGP}-\;N_{Y,Vaccinated}\right)\times {Morb}_{Y-SGP}]$$

Where $N_{Y,Vaccinated\;}$ is the number of young vaccinated. Based on demographic data from the survey, an average of 97.5 % of animals at time of inspection were older than 3 months. Therefore, for the model it was assumed that 97.5 % of all young animals born were vaccinated, as the remaining percentage would be too young at time of vaccination.

### 2.5 Economic modelling to assess disease impact

#### 2.5.1 Farm level SGP financial impact

Annual farm-level gross margin was estimated for each of the production models created (baseline, disease and vaccinated), for both sheep and goats. Gross margin was calculated as follows:

$$Gross\;margin={(N}_{i,offtake,healthy}\times \;P_{i,healthy})+{[N}_{i,SGP,offtake}\times \;P_{i,healthy}\;\times (1-Reduction)]+(N_{i,non-SGP,deaths,}\;\times \;0.05\;\times P_{i,carcass,non-SGP})+(N_{i,SGP,deaths}\;\times 0.05\;\times \;P_{i,carcass,SGP})\;+\Delta Herd\;Value+(N_{replacementsbought\;}\times \;P_{R,f,healthy})-{[N}_{R,0}\;\times (Veterinary\;costs+\;Drug\;costs)]-\;{(N}_{i,offtake\;}\times Market\;fee)-Market\;dues-Market\;travel\;costs-Labour\;costs-\;[(N_{Y\;,\;Vaccinated}+\;N_{R0})\;\times Price\;of\;SGP\;vaccination]-[(N_{i,SGP}-\;N_{i,\;SGP,offtake})\;\times SGP\;treatment\;cost]\;$$

Revenue from offtake of animals was calculated by multiplying offtake by price (P) i.e. sale value of animals. Prices of animals were obtained from the focus group discussion undertaken within the study region. For SGP-affected animals offtaken, the price of healthy animals was multiplied by the price reduction of animals with SGP (Reduction). The number of animal’s dead was multiplied by 0.05, as it was estimated based on authors field experience that farmers will sell 5 % of any animals which have died (due to any cause). For change in herd value (ΔHerd Value), the value of each animal at the beginning and end of the production cycle was calculated. For this, the selling price of animals was used to calculate the value of animals in the herd, and a reduction in value applied for any animals remaining which were SGP-affected. This herd value only incorporates value of the reproductive herd. It is assumed at the start of the annual production cycle that no young animals are in the herd, as animals >1 year old are classed as reproductive. Thus, at the end of the annual production cycle, all young animals have either been offtaken or are now classed as reproductive animals, so valued as such. The number of female replacements $\left(N_{replacementsbought\;}\right)$ purchased in herds with SGP was calculated by multiplying number purchased by sale value.

Veterinary and drug costs per animal were obtained for sedentary herds from a cross-sectional study with 380 respondents in Nigeria (Yesufu et al., 2018) and for transhumance herds from a longitudinal study of 32 pastoralist herds (Majekodunmi et al., 2017). These costs were then multiplied by the reproductive herd size at the beginning of the annual production cycle. Market fee is the cost paid per animal sold at market, comprising of an entrance and a sale fee. Market fees incorporated sales of all animals (both affected and unaffected with SGP). It was assumed that 100 % of animals were sold at market, therefore incurring costs of market fees. This fee was multiplied by the number of animals offtaken over an annual production cycle. Market dues represents a fixed rate paid annually to the market leader, obtained from focus group discussions with small ruminant farmers. Market travel cost is the cost of travelling to market. Labour costs were calculated as the annual salary per herder. This is only applied to transhumance herds, as no paid labour is used in sedentary herds. Focus group discussion identified that on average 1 herder is used per pastoralist herd per production cycle. Price of SGP vaccination was obtained from author estimates, and SGP treatment cost from published research in the study area. For SGP treatment, it was assumed that all SGP-affected animals were treated, apart from reproductive and young animals which were offtaken as a disease coping mechanism. Young SGP-affected animals which were offtaken for the purposes of maintaining the reproductive herd in equilibrium, rather than for SGP disease control were assumed to be treated, as they were offtaken later on in the production cycle.

Subsequently, the results of GM with and without disease were used in a partial budget analysis (PBA) to estimate herd-level SGP financial disease impact in both severely and slightly affected scenarios for a one-year cycle (net SGP cost). Impact of SGP on farmers GM were also calculated by comparing differences in baseline GM’s to GM with SGP disease.

Net SGP cost=(SGP treatment costs+Costs of purchasing replacement females+Offtake of SGP affected animals at reduced price+Reduced value of SGP affected animals remaining in the herd+ SGP mortalities+Reduced number of homebred replacements +Reduced revenue from offtake of young  animals)-(Additonal carcass sold+Reduced marketing costs)

#### 2.5.2 Regional level SGP financial impact

To estimate SGP impact at regional level, the number of herds in each state was firstly calculated. This calculation incorporated author estimates of the proportion of transhumance and sedentary herds within Bauchi, Plateau and Kaduna States. State-level SGP prevalence’s, obtained from the survey, were then utilised to estimate the number of sedentary and transhumance herds that would experience an SGP outbreak over an annual production cycle. Three-year cumulative prevalence rates were obtained for each state, with the annual prevalence rate assumed to be equal for each year; Bauchi 10.00 %, Kaduna 1.00 %, and Plateau 5.67 %. This survey only included sedentary farmers, so prevalence rate calculated was used as a proxy for transhumance herds. The estimated number of affected herds were then multiplied with net cost of SGP at herd-level to obtain an estimation of the overall regional financial disease impact. The net cost of SGP for severely affected herds was utilised for this, as data utilised in the severely affected production models was obtained from previous studies within the region, and no data exists on the likelihood of herds to experience a slight compared to a severe outbreak.

### 2.6 Economic modelling to assess profitability of SGP vaccination strategies

#### 2.6.1 Modelling profitability of vaccination at farm level

To assess the economic efficiency of vaccination at herd-level (net benefit of SGP vaccination), GM of models with disease and unvaccinated and with disease and vaccinated were compared using a PBA. Change in gross margin with SGP vaccination was also calculated by comparing GM’s. A vaccine efficacy of 90 % was utilised as a starting point, obtained from vaccination challenge studies for RM65 and Gorgan strains (EFSA, 2014). Given the uncertainty of this value on the field, vaccine efficacy was explored in the sensitivity analysis in 10 % increments from 10 to 100 % efficacy. Three scenarios of government vaccination subsidies were explored in this herd-level PBA: 0 %, 50 % and 100 %. The 50 % government subsidy scenario was considered the most likely scenario, based on authors experiences with local governments in the region.

Net benefit of SGP vaccination=(Cost of subsidised SGP vaccination+Increased marketing costs+Reduced value of purchased replacements+Reduced value of replacement stock+Reduced revenue from carcass sales+Reduced young animals offtaken )-(Fewer reproductive animals offtaken+Reduced SGP mortality+Increased number of homebred replacements+Fewer young animals offtaken at reduced price+Additional young animals offtaken+Reduced replacement costs+Reduced costs of SGP treatment)

#### 2.6.2 Economic modelling of regional vaccination program

Vaccination strategies modelled were: (1) SGP vaccination only and (2) SGP vaccination incorporated into the annual PPR vaccination programme, to try and understand the possible benefits of a synergy in the vaccination programme. Both strategies encompassed all LGA’s in Bauchi, Kaduna, and Plateau States. In addition, the economic viability of an SGP only programme for an LGA with a high (23.90 %) and low (0.70 %) SGP prevalence were also calculated, in order to directly compare the benefits of vaccinating a high compared to a low risk area, and to see whether a targeted vaccination program is of benefit. For the low-risk and high-risk LGA model, Kanam LGA in Plateau State was utilised, as accurate small ruminant population data was obtained from census data (Adeyinka J. Adedeji, personal comms). In all strategies it was agreed with authors that an overall vaccination coverage of 67.5 % could be achieved, with 75 % of sedentary herds and 60 % of transhumance herds vaccinated.

A CBA of these regional vaccination strategies was undertaken for a 3-year period, and a discount rate of 4.99 % was applied (Central Bank of Nigeria, 2020). Our CBA compared the cost of government investment to the benefits realised by farmers. Thus, we compared the social costs of delivering the vaccination programme to the private benefits realised by farmers. As with the other regional models, this CBA utilised the severely affected model values. It was assumed that annual prevalence rate would be equal for each of the 3 years modelled. For each CBA model, the stochastic model was run for three government subsidies scenarios: 0 %, 50 % and 100 %. Government subsidies were considered as subjective field observations and discussions with partners in Nigeria identified that farmer participation in such programmes would likely increase with government financial support, and to present differing options for policy development. These government subsidies were for the cost of vaccination only, with the remaining costs of delivering the vaccination programme covered entirely by the government.

For each model and subsidy scenario, we calculated both government investment i.e. the subsidised cost of SGP vaccination and costs for delivering the vaccination programme and farmers investment i.e. how much they will pay for the subsidised SGP vaccination. The cost structure for the two regional vaccination programmes and high and low-risk programmes are presented in the results (Table 8). Vaccination cost estimates were obtained from authors, based on their experience with previous PPR mass vaccination campaigns in the region. For SGP with PPR, it was assumed that consumables would be shared, so the only costs accounted for were subsidised cost of vaccine, extra fuel costs, extra staffing costs, sensitisation and cost of consumables required for additional vaccination transportation. An extra 5 days of fuel and staffing costs were estimated to be required per LGA. A full breakdown of the costs of the vaccination programme are presented in Supplementary Information. Benefits of the vaccination programme were based on farmers benefits. For this, the number of farmers who had avoided an outbreak by vaccinating, calculated as the number of herds vaccinated multiplied by the SGP-prevalence rate, was multiplied by the net impact of vaccination at herd-level for severely affected herds. The net present value and BCR of the intervention were calculated for three subsidy scenarios: 0 %, 50 % and 100 %. The cost of investment to the farmer i.e. how much they will pay for the subsidised SGP vaccination was also calculated for each model and subsidy scenario, and this investment cost subtracted from the benefits.

### 2.7 Stochastic simulation and sensitivity analysis

@Risk software for Excel (Palisade Corporation, USA) was utilised to incorporate stochasticity to allow for variability and uncertainty in the model through Monte Carlo stimulations. Programme Evaluation and Review Technique (PERT) or Uniform distribution were fitted to variable or uncertain parameters. Each model was run for 10,000 iterations.

Sensitivity analyses were conducted to assess the effect of individual variables on the model outputs. Advanced sensitivity analyses were undertaken by varying specific disease and economic parameters between their 1 st and 99th distribution percentiles for distributed parameters and varying between −10 % and +10 % from baseline values for non-distributed parameters. Manual sensitivity analysis was additionally undertaken for vaccination efficacy, as this is a critical variable for vaccination economic viability and there is limited field data. Analysis of vaccination efficacy was undertaken assuming subsidised vaccination costs at 50 %, as authors suggested that this was a realistic amount of government funding which could be obtained.

### 2.8 Ethical approval

Ethical approval was granted by the Social Science and Research Ethical Review Board at the Royal Veterinary College (URN SR2020-0266).

## 3 Results

All economic values are presented in pounds sterling (1 ₦ = £0.0019, as consulted on 4th December 2020). Model outputs are presented as median values and their 90 % confidence intervals.

### 3.1 Results from herd production models

Production models developed demonstrated that SGP changed the herd dynamics through increasing number of mortalities and females purchased as replacements and decreasing the numbers of reproductive and young animals offtaken. The model provided a stable reproductive herd size in all scenarios, given our assumption that farmers aim to keep the reproductive herd in equilibrium, with the exception of SGP severely affected, for both sedentary and transhumance herds. A breakdown of these herd dynamics for each of the production models developed are presented in the Supplementary Information.

### 3.2 Farm level gross margins

Results from GM analysis for sedentary herds are shown in Table 4, and for transhumance herds in Table 5. GM was negative in SGP severely affected unvaccinated herds. In sedentary herds, goats represented a greater percentage contribution to the outputs and variable costs, compared to transhumance herds where sheep represented a greater percentage contribution to the outputs and variable costs.

**Table 4:** Gross margin values in £ for sedentary herds for herds without SGP (baseline), SGP severely affected (severe), SGP slightly affected (slight), SGP severely affected and vaccinated (severe – vaccinated), and SGP slightly affected and vaccinated (slight – vaccinated). Overall herd gross margin (GM) and gross margin per reproductive female (GMRF) are from stochastic simulations.

		Baseline	Severe	Slight	Severe – vaccinated	Slight – vaccinated
**OUTPUTS**						
**Animals and products out**	Revenue from sale of healthy reproductive animals	105.36	61.56	95.92	100.98	104.46
	Revenue from sale of SGP-affected reproductive animals	0.00	43.80	9.44	4.38	0.90
	Revenue from sale of healthy young animals	247.39	0.00	186.48	203.55	239.68
	Revenue from sale of SGP-affected young animals	0.00	67.97	26.94	12.77	3.31
	Revenue from sale of carcasses (mortality not due to SGP)	1.58	1.58	1.58	1.58	1.58
	Revenue from sale of carcasses (mortality due to SGP)	0.00	0.43	0.02	0.15	0.01
**Herd value change**	Herd value change	0.00	−147.43	−11.84	−4.89	−1.18
**Animals and products in**	Replacement females	0.00	57.51	0.00	0.00	−0.00
	**Sum of outputs**	**354.33**	**−29.62**	**308.54**	**318.52**	**348.77**
	**Percentage of outputs – sheep (%)**	**30.93**	**58.82**	**30.92**	**30.79**	**30.97**
	**Percentage of outputs – goats (%)**	**69.07**	**41.18**	**69.08**	**69.21**	**69.03**
**VARIABLE COSTS**						
**Veterinary and medicines**	Medicines	17.15	17.15	17.15	17.15	17.15
	Veterinary and Animal Health Workers	0.90	0.90	0.90	0.90	0.90
	SGP treatment	0.00	25.46	4.65	2.97	0.04
	SGP vaccination (50 % subsidies)	0.00	0.00	0.00	2.42	2.42
**Marketing**	Market travel	0.76	0.76	0.76	0.76	0.76
	Annual dues paid to market leader/chief	0.95	0.95	0.95	0.95	0.95
	Market fees	3.89	2.61	3.80	3.91	3.88
	**Sum of variable costs**	**23.65**	**47.83**	**28.21**	**29.05**	**26.10**
	**Percentage of variable costs – sheep (%)**	**36.84**	**33.74**	**34.32**	**34.07**	**34.03**
	**Percentage of variable costs – goats (%)**	**63.16**	**66.26**	**65.68**	**64.93**	**65.97**
	**GM:** Median (90 % CI)	**329 (263−403)**	**−69 (−131 to −4)**	**256 (197−388)**	**290 (230−355)**	**319 (256−388)**
	**GMRF:** 50% subsidies: Median (90 % CI)	**22 (18−26)**^a^	**−5 (−8 to −0.31)**^a^	**17 (13−21)**^a^	**19 (16−23)**	**21 (17−25)**
	**GMRF:** 0 % subsidies: Median (90 % CI)	**22 (18−26)**^a^	**−5 (−8 to −0.31)**^a^	**17 (13−21)**^a^	**18.96 (14.99–23.64)**	**21.01 (17.23–25.35)**
	**GMRF:** 100 % subsidies: Median (90 % CI)	**22 (18−26)**^a^	**−5 (−8 to −0.31)**^a^	**17 (13−21)**^a^	**19.29 (15.39–23.95)**	**21.39 (17.48–25.70)**

a For the baseline, severe and slight scenarios modelled, GMRF does not vary with differing subsidy percentages, as animals are unvaccinated, therefore there is no subsidised vaccination costs.

**Table 5:** Gross margin values in £ for transhumance herds for herds without SGP (baseline), SGP severely affected (severe), SGP slightly affected (slight), SGP severely affected and vaccinated (severe – vaccinated), and SGP slightly affected and vaccinated (slight – vaccinated). Overall herd gross margin (GM) and gross margin per reproductive female (GMRF) are from stochastic simulations.

		Baseline	Severe	Slight	Severe – vaccinated	Slight – vaccinated
**OUTPUTS**						
**Animals and products out**	Revenue from sale of healthy reproductive animals	581.73	458.70	544.56	569.43	579.50
	Revenue from sale of SGP-affected reproductive animals	0.00	123.03	37.17	12.30	2.23
	Revenue from sale of healthy young animals	715.89	0.00	512.61	560.15	688.04
	Revenue from sale of SGP-affected young animals	0.00	178.11	96.90	29.14	10.66
	Revenue from sale of carcasses (mortality not due to SGP)	6.83	6.66	6.82	6.83	6.83
	Revenue from sale of carcasses (mortality due to SGP)	0.00	2.63	0.07	0.42	0.03
**Herd value change**	Herd value change	0.00	−587.97	−74.78	−14.22	−3.55
**Animals and products in**	Replacement females	−0.00	221.03	9.95	4.13	0.00
	**Sum of outputs**	**1304.45**	**−39.88**	**1113.70**	**1159.53**	**1283.74**
	**Percentage of outputs – sheep (%)**	**64.79**	**61.65**	**65.69**	**65.79**	**64.80**
	**Percentage of outputs – goats (%)**	**35.21**	**38.35**	**34.31**	**34.21**	**35.20**
**VARIABLE COSTS**						
**Veterinary and medicines**	Medicines	78.00	78.00	78.00	78.00	78.00
	Veterinary and Animal Health Workers	3.59	3.59	3.59	3.59	3.59
	SGP treatment	0.00	86.07	18.96	10.11	1.82
	SGP vaccination (subsidised)	0.00	0.00	0.00	6.93	6.93
**Labour**	Labour	123.50	123.50	123.50	123.50	123.50
**Marketing**	Market travel	0.95	0.95	0.95	0.95	0.76
	Annual dues paid to market leader/chief	0.76	0.76	0.76	0.76	0.95
	Market fees	9.65	7.96	9.47	8.92	9.62
	**Sum of variable costs**	**216.45**	**300.84**	**235.23**	**232.76**	**134.83**
	**Percentage of variable costs – sheep (%)**	**62.30**	**55.57**	**60.37**	**60.24**	**61.03**
	**Percentage of variable costs – goats (%)**	**37.70**	**44.43**	**39.63**	**39.76**	**38.97**
	**GM:** Median (90 % CI)	**1032 (560−1708)**	**−370 (**−**727 to** −**111)**	**707 (349−1261)**	**877 (459–1479)**	**992 (533–1649)**
	**GMRF:** 50 % subsidies: Median (90 % CI)	**22 (15−29)**^a^	**−8 (−15 to −2)**^a^	**15 (9−22)**^a^	**19.08 (15.28–23.85)**	**21.23 (17.40–25.58)**
	**GMRF:** 0 % subsidies: Median (90 % CI)	**22 (15−29)**^a^	**−8 (−15 to −2)**^a^	**15 (9−22)**^a^	**18.42 (11.91–25.74)**	**20.97 (14.35–28.21)**
	**GMRF:** 100 % subsidies: Median (90 % CI)	**22 (15−29)**^a^	**−8 (−15 to −2)**^a^	**15 (9−22)**^a^	**19.14 (12.19–26.16)**	**21.29 (18.38–28.49)**

a For the baseline, severe and slight scenarios modelled, GMRF does not vary with differing subsidy percentages, as animals are unvaccinated, therefore there is no subsidised vaccination costs.

### 3.3 SGP financial impact at farm and regional level

SGP costs and profit reductions are presented at the herd-level in Table 6. At the herd-level, SGP costs were greatest in transhumance herds. Changes in GM exceeded 100 % in severely affected scenarios, explaining the negative values obtained in GM. In all scenarios, revenue foregone represented the largest proportion of SGP losses (83–92 % of all additional costs due to SGP). In sedentary herds reduced offtake of young animals, as more needed to be kept replacing the reproductive herd, was the greatest contributor to costs (20 %–26 %). In transhumance herds the greatest contributors were reduced price of SGP affected animals offtaken (23 %), and reduction in value of SGP affected animals remaining in the herd (22 %), for slight and severe scenarios respectively.

**Table 6:** Partial budget values in £ for cost of SGP for sedentary (SE) SGP severely affected (severe) and SGP slightly affected (slight) herds and transhumance (TH) SGP severely affected and vaccinated (severe), and SGP slightly affected (slight) herds. Net value (NV), net value per reproductive female (NVRF) and profit loss are from stochastic simulations.

	SE – severe	SE – slight	TH – severe	TH - slight	Explanation
**Additional costs**					
SGP treatment	25.46	4.65	86.07	18.96	All animals affected with SGP, apart from those offtaken, are treated.
Purchase of replacement females	57.52	0.00	221.03	9.95	Purchased as farmers try to maintain female reproductive herd size.
**Revenue foregone**					
Offtake of SGP-affected animals at reduced price	89.96	28.45	379.65	97.66	Reduced value of SGP-affected animals means farmers offtake more reproductive animals to gain the same revenue as without SGP, reducing herd value, and make less revenue from young animal offtake.
Reduced value of SGP-affected animals remaining in herd	72.98	11.84	202.96	74.78	Some SGP-affected reproductive animals remain in the herd, and some reproductive animals are replaced with SGP-affected young homebred stock, which contributes to reduced herd value.
Reduced number of replacements (homebred)	67.27	0.00	373.13	0.00	Reduced numbers of replacement reproductive animals due to increased SGP mortality and offtake contributes to a reduced herd value.
SGP reproductive mortalities	37.35	3.47	158.96	15.47	Some SGP-affected animals die, which could have been sold or remained in the herd.
Fewer young animals offtaken	124.61	12.27	356.21	49.63	Less offtaken as fewer remain after SGP mortality and more kept as replacements.
**Total costs**	**475.15**	**60.24**	**1778.01**	**266.45**	
**Extra Revenue**					
Additional carcasses sold	0.41	0.01	2.45	0.06	Increased mortality due to SGP means there are more carcasses which can be sold.
Value of replacement females (purchased)	57.52	0.00	221.03	9.95	Purchased replacement females contribute to herd value.
Value of additional replacement stock (homebred)	7.82	10.21	62.88	46.43	For some sub-populations, need for increased homebred replacements can be met, so there are more than in herds without SGP, contributing to herd value.
Additional young offtaken	0.00	0.00	61.24	0.00	Applies to transhumance severely affected herds, where offtake of SGP-affected animals as a coping mechanism results in more female sheep being sold.
**Costs saved**					
Reduced marketing costs	1.28	0.09	1.68	0.18	Selling fewer young animals results in reduced marketing costs.
**Total benefits**	**67.03**	**10.31**	**349.29**	**56.62**	
**NV:** Median (90 % CI)	**404 (479−323)**	**71 (109−43)**	**1409 (2236−838)**	**303 (579−147)**	
**NVRF:** Median (90 % CI)	**27 (31−22)**	**5 (7−3)**	**30 (41−21)**	**7 (10−3)**	
**GM reduction (%):** Median (90 % CI)	**121 (143−101)**	**22 (32−13)**	**138 (174−109)**	**31 (47−16)**	

At the regional-level, median SGP disease cost was greatest in Bauchi State: £19.9 million (90 % CI: £26 million, £5.1 million), followed by Plateau State: £3.8 million (90 % CI: £5.4 million, £0.3 million) and Kaduna State: £0.8 million (90 % CI: £1.5 million, £0.9 million). Aggregated median regional costs estimated were £24.5 million (90 % CI: £31.9 million, £18.6 million).

### 3.4 Economic profitability of SGP vaccination strategies at herd and regional level

SGP vaccination benefits at the herd-level are shown in Table 7. SGP vaccination resulted in herd-level net benefits in both herd types and disease scenarios. Benefits of vaccination were greatest in transhumance herds. Subsidised SGP vaccination cost represented between 1.9–20.8 % of total costs to farmers. Vaccination cost structure of the strategies explored is shown in Table 7, and CBA results for SGP vaccination at the regional level shown in Table 9. At the regional-level, vaccination was found to be profitable when comparing government costs to societal benefits, over three-years for both strategies explored, with greater BCR’s realised when combining SGP vaccination with the PPR programme. Net present value and BCR was greatest in high-risk compared to low-risk LGA, with benefits only realised in low-risk LGA’s when costs of the SGP vaccination is 100 % subsidised by the government.

**Table 7:** Partial budget values in £ for benefits of SGP vaccination for vaccinated sedentary (SE) SGP severely affected (severe) and SGP slightly affected (slight) herds and vaccinated transhumance (TH) SGP severely affected (severe), and SGP slightly affected (slight) herds. Net value (NV), net value per reproductive female (NVRF) and benefit: cost ratio (BCR) are from stochastic simulations.

	Severe - SE	Slight - SE	Severe – TH	Slight - TH	Explanation
**Additional costs**					
SGP vaccination (50 % subsidies)	2.42	2.42	6.93	6.93	In this scenario government subsidises 50 % of the costs of the vaccine.
Increased marketing costs	1.29	0.00	0.95	0.15	Reduced SGP mortality and reproductive replacement rate, so more offtaken.
**Revenue foregone**					
Reduced value of female replacements (purchased)	57.51	0.00	216.91	9.95	Fewer mortalities and offtake due to SGP, so farmers do not always have to purchase females as replacements, they can achieve this using homebred stock.
Reduced replacement stock (homebred)	7.04	9.13	54.73	43.03	Number of replacement homebred stock decreases, as less are needed for replacement of reproductive herd, contributing to reduced herd value.
Fewer carcasses sold	0.26	0.01	2.03	0.03	Reduced mortality, so reduced carcasses to sell.
Reduced young animals offtaken	0.00	0.00	85.99	0.00	Fewer female young sheep offtaken as less SGP-affected.
**Total costs**	**68.52**	**60.24**	**367.54**	**60.09**	
**Extra Revenue**					
Fewer reproductive animals offtaken	31.65	6.07	123.15	38.44	As fewer animals are SGP-affected and therefore sold at a lower value, farmers sell less animals than in unvaccinated herds to generate the same revenue.
Reduced SGP reproductive mortality	33.61	3.11	143.06	14.54	Vaccination reduces morbidity and therefore mortality.
Reduced SGP-affected animals remaining in herd	74.04	10.66	173.64	70.93	Fewer animals are SGP-affected, therefore fewer remain in the herd.
Increased replacement stock (homebred)	73.74	0.00	390.44	0.00	Reduced SGP mortality and offtake in young herd, increasing replacements for most sub-populations.
Fewer young animals offtaken at reduced price	48.47	18.68	210.24	44.95	Fewer SGP-affected young animals so fewer sold at lower value.
Additional young animals offtaken	99.88	10.89	286.93	44.34	Less young animals are required for replacement of reproductive herd, and fewer die due to SGP, so more are available to offtake.
**Costs saved**					
Reduced replacement females purchased	57.51	0.00	216.91	9.95	Reduced costs of purchasing reproductive females for breeding, as fewer are purchased.
Reduced cost of SGP treatment	22.49	4.61	75.96	17.14	Fewer animals are SGP-affected, therefore fewer are treated.
**Total benefits**	**441.39**	**54.02**	**1620.33**	**240.19**	
**NV:** 50 % subsidies: Median (90 % CI)	**358 (239−431)**	**59 (35−92)**	**1251 (737−2017)**	**264 (124−516)**	
**NVRF:** 50 % subsidies: Median (90 % CI)	**23.76 (19.28−28.61)**	**4.01 (2.36−6.31)**	**26.85 (17.99−37.02)**	**7.45 (3.47−15.14)**	
**NVRF:** 0 % subsidies: Median (90 % CI)	**23.58 (19.21–28.49)**	**2.77 (2.20−6.13)**	**26.71 (17.81–36.85)**	**7.43 (3.37–14.90)**	
**NVRF:** 100 % subsidies: Median (90 % CI)	**23.95 (19.42–28.76)**	**4.35 (2.53–6.46)**	**27.01 (18.07–37.19)**	**7.58 (3.68–15.37)**	
**BCR:** 50 % subsidies: Median (90 % CI)	**6.62 (5.3−8.9)**	**5.01 (3.63−7.62)**	**5.27 (3.31−15.08)**	**5.06 (2.75−14.01)**	
**BCR:** 0 % subsidies: Median (90 % CI)	**6.39 (5.14–8.43)**	**4.37 (3.18–6.27)**	**5.14 (3.31–13.81)**	**4.61 (2.64–11.33)**	
**BCR:** 100 % subsidies: Median (90 % CI)	**6.87 (5.49–9.35)**	**6.11 (4.24–10.1)**	**5.37 (3.31–15.24)**	**5.23 (2.88–17.60)**

**Table 8:** Vaccination cost structure in £ for 3 years for the vaccination strategies explored: Regional SGP only, Regional SGP and PPR, and for an LGA (high and low risk) at 50 % vaccination subsidies.

	Regional SGP	Regional SGP and PPR	LGA (high and low risk)^a^	Explanation
**YEAR 1**				
Vaccine (subsided cost)	441,167	441,167	24,904	Government subsidises the cost of the vaccine. In this example, a 50 % government subsidy was used.
Consumables	37,981	3,667	993	Includes cold boxes, alcohol, cotton wool, gloves, disposable lab coats, disinfectant, detergent, rubbish/sharps cans, syringes, and needles, first aid, ice packs, hand soap, hand sanitiser, boots, stationery, face mask, storage box and fuel. For the combined SGP and PPR programme, fuel was the only consumable considered, as it was assumed that other consumables were shared from the PPR programme.
Staffing	90,772	34,770	1807	For one veterinarian, two vaccinators and a driver.
Sensitisation	15,504	15,504	190	To cover the costs of LGA staff and posters/radio/other forms of advertisement to engage local community.
**Year 1 cost**	**585,424**	**495,108**	**27,893**	
**YEAR 2**				
Vaccine (subsidised cost)	441,167	441,167	24,904	Remains the same as the same number of animals are vaccinated.
Consumables	37,734	3420	746	Storage boxes, ice packs and cold boxes are not included, as only an initial purchase is required.
Staffing	90,772	34,770	1807	Same staffing requirements.
Sensitisation	15,504	15,504	190	Same sensitisation requirements.
**Year 2 cost**	**585.177**	**495,108**	**27,646**	
**YEAR 3**				
Vaccine (subsidised cost)	441,167	441,167	24,904	Remains the same as the same number of animals are vaccinated.
Consumables	37,734	3420	746	Storage boxes, ice packs and cold boxes are not included in this, as only an initial purchase is required.
Staffing	90,772	34,770	1807	Same staffing requirements.
Sensitisation	15,504	15,504	190	Same sensitisation requirements.
**Total cost – Year 3**	**585,177**	**495,108**	**27,646**	
**TOTAL COST OF PROGRAMME**	**1,755,779**	**1,484,829**	**83,186**	

a Vaccination costs are presented together for both high and low-risk LGA’s, as the vaccination costs remain the same for the two scenarios as the same number of animals are vaccinated.

**Table 9:** Net present value (NPV) and benefit to cost ratio (BCR) in £ from stochastic simulations, farmers investment and government investment for regional sheep and goat pox (SGP) vaccination strategies and high and low-risk states over 3-years.

	NPV	BCR	Farmers investment	Government investment
	Median (90 % CI)	Median (90 % CI)		
**SGP vaccination only**				
0 % subsidies	32,117,731 (22,518,836–44,138,951)	27 (19−36)	6,254,783	1,250,038
50 % subsidies	33,216,231 (23,707,516−45,095,420)	11 (8−15)	3,127,391	2,031,471
100 % subsidies	34,369,745 (24,657,350−46,403,760)	6 (5−8)	0	6,647,970
**SGP vaccination with PPR program**				
0 % subsidies	33,221,284 (23,622,390–45,242,505)	228 (162–310)	6,254,783	146,485
50 % subsidies	33,227,197 (23,742,248−45,052,010)	11 (8−15)	3,127,391	1,348,116
100 % subsidies	33,295,761 (23,653,894–45,256,927)	7 (6−10)	0	6,401,268
**High-risk LGA**				
0 % subsidies	3,121,331 (2,595.129–4,292,748)	439 (338–558)	168,678	7705
50 % subsidies	3,246,399 (2,487,262−4,147,529)	36 (29−45)	84,399	75,538
100 % subsidies	3,318,767 (2,353,868–4,005,165)	19 (15−23)	0	176,384
**Low-risk LGA**				
0 % subsidies	91,379 (68,528−118,249)	13 (10−16)	168,678	7705
50 % subsidies	−76,406 (−99.252 to −51,793)	0.17 (0.05−0.43)	84,399	75,538
100 % subsidies	−243,995 (−278,964 to −209.808)	−0.38 (−0.24 to −0.50)	0	176,384

### 3.5 Sensitivity analysis

**Fig. 3:**
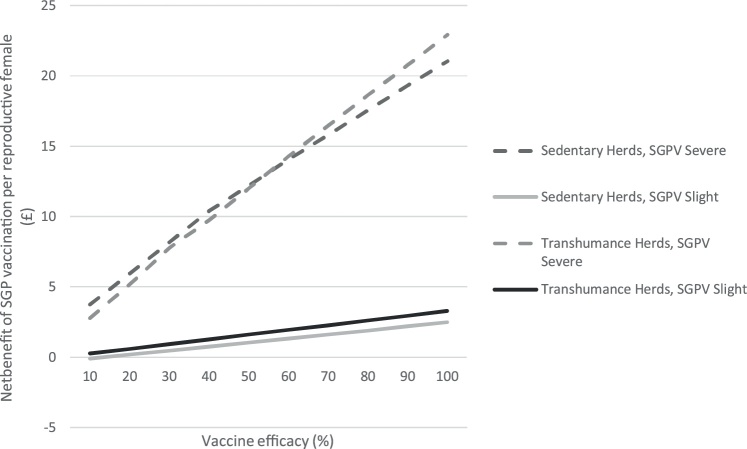
Results from manual sensitivity analysis, varying vaccine efficacy between 10–100 % and recording effect on net benefit per reproductive female at the herd-level, with government vaccination subsidies at 50 %.

Sensitivity analysis for vaccination efficacy at herd-level shows SGP vaccination realises a positive benefit with vaccination efficacy between 10–100 % and government subsidies at 50 %. The exception was in slightly affected sedentary herds, where a slight loss of £0.10 per reproductive female occurs when vaccine efficacy is 10 % (Fig. 3).

**Fig. 4:**
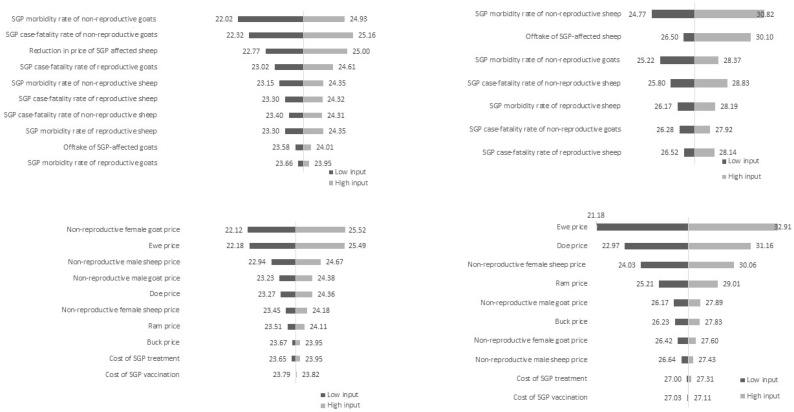
Tornado graphs from advanced sensitivity analysis undertaken for the net cost of SGP in severely affected herds for SGP epidemiological parameters (top) and economic parameters (bottom) for sedentary herds (left) and transhumance herds (right).

Advanced sensitivity analysis on disease models showed that for sedentary herds, the most influential epidemiological parameter is SGP morbidity rate of young goats, and price of young female goats the most influential economic parameter. For transhumance herds, SGP morbidity rate of young sheep is the most influential epidemiological parameter, and ewe price the most influential economic parameter. The tornado graphs in Fig. 4 for net benefit of SGP vaccination per reproductive female show that with high and low inputs, a positive net benefit is still realised.

## 4 Discussion

This study estimated costs and assessed economic profitability of vaccination for SGP in subsistence farmers in Northern Nigeria, economic tools with epidemiological data to understand impact of policy decisions surrounding SGP vaccination. By estimating costs and benefits of different SGP vaccination scenarios, this study is a step forward from previous economic studies of SGP in Northern Nigeria and aims to inform policy development and create incentive for future investment in SGP control in the area.

SGP costs estimated suggest significant losses for subsistence farmers. Transhumance herds have greater herd-level loss compared to sedentary herds arising from the different management, disease coping strategies and SGP epidemiology. This includes a higher rate of offtake of SGP-affected animals at a lower cost, higher morbidity rates in young goats and higher case-fatality rate in young sheep in transhumance herds. Estimated costs were £93 and £168 greater for sedentary and transhumance herds, respectively, when compared to herd-level loss previously calculated in Bauchi (Limon et al., 2020). These differences can be attributed to different methodologies; developing production and GM models enabled comparisons between infected and baseline herds, so costs arising from changes in population dynamics, such as reduced number of replacements from own stock could be estimated, which previous literature within the study area did not quantify (Bolajoko et al., 2019; Limon et al., 2020). Given their selection criteria, previous studies in the region are considered to only include severely affected herds. In this study slight and severe SGP scenarios were considered for herd-level modelling based on observations in the field. However, these differences across SGP affected herds have not been formally evaluated and therefore the slightly affected scenario considered in this study is theoretical. Future studies should be planned to better understand and formally quantify different levels of severity in affected herds. Top contributors to SGP cost identified in PBA (mortality, offtaking animals at reduced price, and reduction in affected animals remaining in herd), and the most influential parameters for SGP cost identified in sensitivity analysis (SGP case-fatality rate and morbidity rate), are aligned with results from previous studies (Garner et al., 2000; Senthilkumar and Thirunavukkarasu, 2010; Ben Chehida et al., 2018). Additionally, this study identified reduction in numbers of young animals offtaken as a large contributor to costs which has not previously been reported. Impacts of SGP on herd-level dynamics identified the reduction in reproductive herd size at the end of the year in severely affected herds, suggesting potential longer-term economic effects of SGP. It is acknowledged that our model reflected current values of animals and disease coping practices of small ruminant farmers, however, in the long-term SGP disease outbreaks may have dynamic effects, such as increasing prices of healthy animals, altering the costs of SGP to farmers.

Across the three states it was estimated based on our assumptions that 33,204 flocks would have an SGP outbreak over the annual production cycle in the absence of vaccination with total regional costs estimates exceeding £24 million. The greatest losses occurred in Bauchi, as it has the highest SGP - prevalence rate and small ruminant population of the three states explored in this study. SGP cost at the regional level has not previously been reported in Nigeria. Previously, transhumance herds have been reported to have a lower prevalence rate of SGP than sedentary herds (Limon et al., 2020) due to increased mixing and higher stocking densities, therefore regional costs of SGP for transhumance herds may be overestimated as we used SGP-prevalence rates for sedentary herds as a proxy for transhumance herds in this study. Adjusting for different numbers of flocks estimated to be affected in each study, our estimates exceed that calculated in previously in India with similar small ruminant production systems (Garner et al., 2000). This difference is likely to be due to assumptions made in our modelling on disease coping mechanisms, geographical economic factors, and husbandry factors. In the study in India 65 % of participants had goats only in their herds, 5 different herd types were considered for analysis and two cost scenarios were considered regionally; farmers familiar with SGP whom incurred lower SGP costs and those unfamiliar with SGP whom incurred higher SGP costs.

Assessment of economic viability of SGP vaccination determined a net benefit; with a positive BCR and NVRF realised for each of the subsidy scenarios considered. Given the assumptions considered, benefits outweighed costs of vaccination by £372.87 and £42.46 in sedentary herds and £1752.79 and £180.10 in transhumance herds at 50 % subsidy levels, for the severely and slightly affected scenario respectively. SGP vaccination is currently not commercially available in Nigeria and the potential financial benefits of subsistence farmers vaccinating their herds have not been quantified before either in Nigeria or other nearby endemic settings. Sensitivity analysis demonstrated that benefit is realised at the herd-level even with low vaccination efficacy. Benefits for farmers are realised also with the regional-vaccination strategies proposed. Greater BCR’s observed in high-risk areas suggest that targeting these areas would result in a larger value for investment and would be less costly and more straightforward to implement. For all regional vaccination scenarios considered, the cost of the SGP vaccine represented the highest cost of the programme. High costs of the vaccine explain why combining SGP vaccination with PPR vaccination realises little difference in BCR and net present value as consumables and staffing represent a small investment proportion. High costs of vaccination have also been estimated for other small ruminant diseases in Northern Nigeria, with PPR vaccination estimated to cost just over £21.5 million when considering the costs of vaccine alone (Fadiga et al., 2013). As SGP vaccination is not commercially available within Nigeria currently, our estimates on vaccination costs were based on authors experience, these could be lower once the vaccine is available. At the herd-level, vaccination cost had little influence on benefits of SGP vaccination with 50 % subsidies.

Our study utilised integrated herd and economic models; integrating such models is considered a reliable method of estimating disease impact and exploring the potential profitability of disease control programmes. Such models had not previously been used to explore the economic impact of animal diseases or their vaccination in Nigeria. Our method focused solely on the benefits of SGP vaccination for farmers and did not account for the potential externalities of such vaccination programmes and the impact of these. Wide-spread implementation of SGP vaccination will increase livestock productivity, with potential impacts on market dynamics; vaccination will increase supply and thus potentially reduce prices of animals, so benefits of the program may this way be overestimated. Economic surplus analysis is recommended for future studies to more accurately assess the benefits of these programs. Undertaking a time series analysis (Barratt et al., 2019) would also enable the exploration of impact of SGP vaccination on markets. However, both these methods are limited in their requirement for large amounts of current and historic data on markets and prices, which are not available within the study region.

Our economic analyses only considered direct losses and benefits of SGP and its vaccination to farmers, excluding indirect effects and wider societal benefits such as impact on trade. Therefore, SGP costs and vaccination benefits to farmers are likely underestimated. The framework of our CBA could be altered to incorporate such benefits, to calculate a total social-cost benefit. Economic analysis to explore the benefits for governments and other stakeholders within the supply chain, such as vaccination manufacturers, would potentially create more incentive to invest. Conducting a feasibility analysis; comparing analysis of other SGP control methods, such as enhanced surveillance, or an opportunity cost analysis would also be beneficial.

Data parameterisation of models identified lack of some data values within the literature, particularly around costs such as price of young sheep and goats, highlighting the need for further research and systematic data collection within Nigeria. Where parameters were unavailable in the literature, they were obtained from authors’ experience or from focus group discussion with farmers. Such focus groups introduced a potential recall or reporting bias. Only small number of farmers were invited to take part and participation was voluntary, bringing into question the representativeness of economic data collected in these discussions. Nevertheless, greater value for transhumance compared to sedentary animal prices corresponds with reports from the area (Limon et al., 2020). Lack of SGP-prevalence rates for transhumance herds meant that the same rates of sedentary farmers was utilised. Another demographic of farmers who are traders is known to exist within the region, but they were not considered in this study due to data and time constraints.

Our models are based on numerous assumptions, however, assumptions used were obtained from either field observations by partners or authors working in Nigeria, so were considered to reflect common practices, or from literature in Nigeria or similar settings. The assumptions used in this model are an additional useful output in this study; highlighting areas of uncertainty and requirements for further key data collection and research to improve model reliability and knowledge of animal husbandry, SGP disease reaction and SGP epidemiology. Collecting such data can help optimise industry and government investment. Given our models basis on numerous assumptions and variable parameters, uncertainty existed in modelling; demonstrated by the wide confidence intervals obtained for outputs. However, our sensitivity analyses demonstrated that low and high inputs of variable parameters resulted in net SGP cost and benefit from vaccination. The model developed in this study can be easily updated once data gaps identified are addressed and there is further understanding of SGP epidemiology and disease dynamics within the study area. Similarly, the models can be adapted to incorporate other production systems such as trader-farmers.

### 4.1 Conclusion

This study provides further evidence of the significant economic burden SGP represents to subsistence farmers within Northern Nigeria. SGP vaccination was determined to be economically viable at the herd and regional level for most scenarios considered, providing a net benefit for both sedentary and transhumance herds. The findings of this research can contribute policy discussions on implementing an economically viable SGP vaccination programme, and models developed can be used to explore financial impact of other small ruminant diseases within Northern Nigeria.

## Funding

This work contributed to the project “Development of rationally designed live-attenuated lumpy skin disease vaccines” BB/R008833 led by The Pirbright Institute. The Pirbright Institute receives strategic funding support from Biotechnology and Biological Science Research Council, United Kingdom (BBS/E/I/00007031, BBS/E/I/00007036, BBS/E/I/00007037, and BBS/E/I/00007039). PMB was partially funded by BBSRC-funded Institute Strategic Programmes BB/J004324/1 and BBS/E/D/20002173 awarded to The Roslin Institute.

## Declaration of Competing Interest

The authors report no declarations of interest.
