# Supplementary material for: Financial impact of sheeppox and goatpox and estimated profitability of vaccination for subsistence farmers in selected northern states of Nigeria
Source: Prev Vet Med. 2022 Jan;198:105503. doi: 10.1016/j.prevetmed.2021.105503 (PMC8784823; doi:10.1016/j.prevetmed.2021.105503)
Supplement: Supplementary file 1 [file mmc1.docx]

**Supplementary Table 1.** Description of the small ruminant production system types in Northern Nigeria considered for this study

| **System** | **Description** |
| --- | --- |
| Transhumance (Pastoral) | Extensive management of small ruminants, often alongside cattle. Ruminants are reared alongside low-levels of crop-cultivation. A permanent home-base exists but seasonal herd movement for resource optimisation occurs, driven by climatic conditions. Goats are often sedentary, remaining at the home-base with female members of the family during seasonal herd movement. Livestock production is often a primary source of income in these systems, so high offtake rates are observed as a means of income generation. Characterised by larger herd sizes and a higher proportion of sheep than in sedentary systems. 50-65% of herds within the region are estimated to be transhumance herds. |
| Sedentary (Backyard) | Extensive management of small ruminants, most commonly goats. Communal grazing is used, and small ruminants are housed overnight. Grazing is often supplemented by left-over food and excess forage, especially during the dry season. Livestock production often serves as a secondary income source in these systems. 35-50% of herds within the region are estimated to be sedentary herds. |

###

**Supplementary Table 2.** Herd production dynamics over an annual production cycle for sedentary herds (SE) without SGP (baseline), SGP severely affected (severe), SGP slightly affected (slight), SGP severely affected vaccinated (severe - vaccinated) and SGP slightly affected (slight - vaccinated), from herd production models.

|  |  | **SE – baseline** | **SE – severe** | **SE – slight** | **SE – severe - vaccinated** | **SE – slight - vaccinated** |
| --- | --- | --- | --- | --- | --- | --- |
| Herd size beginning | Sheep | 7 | 7 | 7 | 7 | 7 |
|  | Goats | 12 | 12 | 12 | 12 | 12 |
| Number of births | Sheep | 9 | 8 | 9 | 9 | 9 |
|  | Goats | 24 | 24 | 24 | 24 | 24 |
| Number of SGP-affected animals | Sheep | 0 | 10 | 1 | 0 | 0 |
|  | Goats | 0 | 20 | 3 | 2 | 0 |
| Number of mortalities (reproductive) | Sheep | 0 | 1 | 0 | 0 | 0 |
|  | Goats | 1 | 3 | 2 | 2 | 1 |
| Number of mortalities (young) | Sheep | 2 | 4 | 2 | 2 | 2 |
|  | Goats | 8 | 14 | 8 | 8 | 8 |
| Number of animals offtaken (reproductive) | Sheep | 1 | 2 | 1 | 1 | 1 |
|  | Goats | 3 | 4 | 3 | 3 | 3 |
| Number of animals offtaken (young) | Sheep | 4 | 2 | 4 | 4 | 4 |
|  | Goats | 12 | 6 | 11 | 11 | 12 |
| Number of purchased replacements into herd (females) | Sheep | 0 | 1 | 0 | 0 | 0 |
|  | Goats | 0 | 1 | 0 | 0 | 0 |
| Herd size end | Sheep | 7 | 6 | 7 | 7 | 7 |
|  | Goats | 12 | 10 | 12 | 12 | 12 |

**Supplementary Table 3.** Herd production dynamics over an annual production cycle for transhumance herds (TH) without SGP (baseline), SGP severely affected (severe), SGP slightly affected (slight), SGP severely affected vaccinated (severe - vaccinated) and SGP slightly affected (slight - vaccinated), from herd production models

|  |  | **TH – baseline** | **TH – severe** | **TH – slight** | **TH – severe – vaccinated** | **TH – slight - vaccinated** |
| --- | --- | --- | --- | --- | --- | --- |
| Herd size beginning | Sheep | 38 | 38 | 38 | 38 | 38 |
|  | Goats | 23 | 23 | 23 | 23 | 23 |
| Number of births | Sheep | 41 | 40 | 41 | 41 | 41 |
|  | Goats | 46 | 45 | 46 | 46 | 46 |
| Number of SGP-affected animals | Sheep | 0 | 30 | 8 | 3 | 0 |
|  | Goats | 0 | 45 | 6 | 5 | 0 |
| Number of mortalities (reproductive) | Sheep | 6 | 9 | 6 | 6 | 6 |
|  | Goats | 4 | 6 | 4 | 4 | 4 |
| Number of mortalities (young) | Sheep | 12 | 24 | 12 | 14 | 12 |
|  | Goats | 14 | 28 | 16 | 16 | 14 |
| Number of animals offtaken (reproductive) | Sheep | 10 | 13 | 11 | 10 | 10 |
|  | Goats | 7 | 10 | 7 | 7 | 7 |
| Number of animals offtaken (young) | Sheep | 12 | 8 | 11 | 10 | 12 |
|  | Goats | 22 | 12 | 20 | 19 | 21 |
| Number of purchased replacements into herd (females) | Sheep | 0 | 6 | 0 | 0 | 0 |
|  | Goats | 0 | 3 | 0 | 0 | 0 |
| Herd size end | Sheep | 38 | 31 | 38 | 38 | 38 |
|  | Goats | 23 | 14 | 23 | 23 | 23 |

| Supplementary Table 4. Production and economic parameters and their distributions used in stochastic simulation for sedentary herds. SGP epidemiological and vaccination parameters are presented in Table 2 of the main text. | | | | |
| --- | --- | --- | --- | --- |
|  | Value | Range^1^ | Reference/Calculation | Distribution used in stochastic simulation |
| PRODUCTION PARAMETERS |  |  |  |  |
| *Goats* |  |  |  |  |
| Offtake rate – does (proportion) | 0.2285 | 0.167-0.29 | (Ogah, 2018; Otte and Chilonda, 2002) | Uniform |
| Offtake rate – bucks (proportion) | 0.2285 | 0.167-0.29 | (Ogah, 2018; Otte and Chilonda, 2002) | Uniform |
| Mortality rate – does (proportion) | 0.15 | 0.1-0.2 | (Ameh et al., 2000; Bayer, 1983; Otte and Chilonda, 2002; Sumberg and Cassaday, 1985) | Uniform |
| Mortality rate – bucks (proportion) | 0.144 |  | (Bayer, 1983; Sumberg and Cassaday, 1985) | Non-distributed |
| Replacement rate – does (proportion) | 0.3785 |  | Sum of offtake and mortality | Dependent on distributed parameters (offtake and mortality) |
| Replacement rate – bucks (proportion) | 0.3725 |  | Sum of offtake and mortality | Dependent on distributed parameters (offtake and mortality) |
| Parturition rate/doe/year | 1.68 | 1.34-2.1 | (Ajala et al., 2008; Ogah, 2018; Seybolt, 2015; Sumberg and Cassaday, 1985) | Uniform |
| Prolificacy | 1.5 | 1.3-1.7 | (Adu et al., 1979; Ajala, MK, Lamidi, OS, Otaru, 2008; Bayer, 1983; Ogah, 2018; Sumberg and Cassaday, 1985) | Uniform |
| Mortality rate – non reproductive female goats (proportion) | 0.275 | 0.22-0.331 | (Bayer, 1983; Karen Ann Dvorak, 1993; Sumberg and Cassaday, 1985) | Uniform |
| Mortality rate – non reproductive male goats (proportion) | 0.275 | 0.22-0.331 | (Bayer, 1983; Karen Ann Dvorak, 1993; Sumberg and Cassaday, 1985) | Uniform |
| Herd size | 6 | 0-6 | (Georgina, Unpublished) | PERT |
| Proportion female | 0.8 |  | Expert opinion | Non-distributed |
| *Sheep* |  |  |  |  |
| Offtake rate – ewes (proportion) | 0.238 | 0.156-0.32 | (Ogah, 2018; Otte and Chilonda, 2002) | Uniform |
| Offtake rate – rams (proportion) | 0.238 | 0.156-0.32 | (Ogah, 2018; Otte and Chilonda, 2002) | Uniform |
| Mortality rate – ewes (proportion) | 0.075 |  | (Otte and Chilonda, 2002) | Non-distributed |
| Mortality rate – rams (proportion) | 0.075 |  | (Otte and Chilonda, 2002) | Non-distributed |
| Ewe replacement rate (proportion) | 0.313 |  | Sum off offtake and mortality | Dependent on distributed parameters (offtake and mortality) |
| Ram replacement rate (proportion) | 0.313 |  | Sum of offtake and mortality | Dependent on distributed parameters (offtake and mortality) |
| Parturition rate/ewe/year | 1.24 | 0.88-1.6 | (Ajala et al., 2008; Ogah, 2018; Seybolt, 2015; Sumberg and Cassaday, 1985) | Uniform |
| Prolificacy | 1.23 |  | (Ogah, 2018; Seybolt, 2015) | Non-distributed |
| Mortality rate – non reproductive female sheep (proportion) | 0.266 |  | (Otte and Chilonda, 2002) | Non-distributed |
| Mortality rate – non reproductive male sheep (proportion) | 0.266 |  | (Otte and Chilonda, 2002) | Non-distributed |
| Herd size | 14 | 8-16 | (Adeyinka J.Adedeji, Manuscript in preparation) | PERT |
| Proportion female | 0.8 |  | Authors opinion | Non-distributed |
| ECONOMIC PARAMETERS (£) |  |  |  |  |
| *Goats* |  |  |  |  |
| Value of healthy doe | 26.60 | 24.70-28.50 | Field data from focus group discussions undertaken by authors in Nigeria. | Uniform |
| Value of healthy buck | 6.65 | 1.90-11.40 | As above. | Uniform |
| Value of healthy young female goat | 18.05 | 7.60-28.50 | As above. | Uniform |
| Value of healthy young male goat | 13.30 | 11.40-15.20 | As above. | Uniform |
| Value of healthy doe carcass | 3.99 |  | Value of carcass is between 10-20% of value of healthy animal. 15% reduction was utilised in the deterministic model (Authors experience). | Dependent on distributed parameter (value of animals) |
| Value of healthy buck carcass | 1.00 |  | As above. | Dependent on distributed parameter (value of animals) |
| Value of healthy young female carcass | 2.71 |  | As above. | Dependent on distributed parameter (value of animals) |
| Value of healthy young male carcass | 2.00 |  | As above. | Dependent on distributed parameter (value of animals) |
| Value of SGP affected doe | 14.67 |  | SGP affected goats are sold with a 30.00-59.17% reduction in price, arising from clinical manifestations of disease including weight loss, dyspnoea and skin lesions A mean reduction off 44.85% was used in the deterministic model (Bolajoko et al., 2019; Limon et al., 2020) | Dependent on distributed parameter (value of animals) |
| Value of SGP affected buck | 3.67 |  | As above. | Dependent on distributed parameter (value of animals) |
| Value of SGP affected young female goat | 9.95 |  | As above. | Dependent on distributed parameter (value of animals) |
| Value of SGP affected young male goat | 7.33 |  | As above. | Dependent on distributed parameter (value of animals) |
| Value of SGP affected doe carcass | 1.20 |  | Value of carcass is between 10-20% of value of healthy animal. 15% reduction was utilised in the deterministic model (Authors experience). | Dependent on distributed parameter (value of animals) |
| Value of SGP-affected buck carcass | 0.30 |  | As above. | Dependent on distributed parameter (value of animals) |
| Value of SGP-affected young female carcass | 0.81 |  | As above. | Dependent on distributed parameter (value of animals) |
| Value of SGP-affected young male carcass | 0.60 |  | As above. | Dependent on distributed parameter (value of animals) |
| *Sheep* |  |  |  |  |
| Value of healthy ewe | 26.60 | 15.20-38.00 | Field data from focus group discussions undertaken by authors in Nigeria.. | Uniform |
| Value of healthy ram | 23.75 | 13.30-34.20 | As above. | Uniform |
| Value of healthy young female sheep | 16.15 | 7.60-24.70 | As above. | Uniform |
| Value of healthy young male sheep | 16.15 | 9.50-22.80 | As above. | Uniform |
| Value of healthy ewe carcass | 3.99 |  | Value of carcass is between 10-20% of value of healthy animal. 15% reduction was utilised in the deterministic model (Authors Opinion). | Dependent on distributed parameter (value of animals) |
| Value of healthy ram carcass | 3.56 |  | As above. | Dependent on distributed parameter (value of animals) |
| Value of healthy young female sheep carcass | 2.42 |  | As above. | Dependent on distributed parameter (value of animals) |
| Value of healthy young male carcass | 2.42 |  | As above. | Dependent on distributed parameter (value of animals) |
| Value of SGP affected ewe | 14.90 |  | SGP affected goats are sold with a 30.00-59.17% reduction in price, arising from clinical manifestations of disease including weight loss, dyspnoea and skin lesions A mean reduction off 44.85% was used in the deterministic model (Bolajoko et al., 2019; Limon et al., 2020) | Dependent on distributed parameter (value of animals) |
| Value of SGP affected ram | 13.30 |  | As above. | Dependent on distributed parameter (value of animals) |
| Value of SGP affected young female sheep | 9.04 |  | As above. | Dependent on distributed parameter (value of animals) |
| Value of SGP affected young male sheep | 9.04 |  | As above. | Dependent on distributed parameter (value of animals) |
| Value of SGP affected ewe carcass | 1.20 |  | SGP affected carcass are sold with a 70-90% reduction in value, arising from poorer carcass quality due to damage to hide and skins. A median of 80% was used in the deterministic model. (Bolajoko et al., 2019) | Dependent on distributed parameter (value of animals) |
| Value of SGP affected ram carcass | 1.07 |  | As above. | Dependent on distributed parameter (value of animals) |
| Value of SGP affected young female sheep carcass | 0.73 |  | As above. | Dependent on distributed parameter (value of animals) |
| Value of SGP affected young male sheep carcass | 0.73 |  | As above. | Dependent on distributed parameter (value of animals) |
| *Veterinary and medicine costs* |  |  |  |  |
| Drug costs/animal | 0.90 |  | A study in Nigeria found a total annual cost of veterinary and drugs as £31 for a sedentary goat herd with 31 animals. In transhumance herds, 95% of veterinary and drug costs is on drugs and 5% on veterinary services, so this was assumed for sedentary herds also (Majekodunmi et al., 2017). This figure was also applied to sheep, as there was no data on veterinary and drug costs for sedentary sheep in Nigeria in the literature.  This value was agreed with authors to be a reasonable average cost in sedentary herds during discussions. | Non-distributed |
| Veterinary costs/  animal | 0.05 |  | A study in Nigeria found a total cost of veterinary and drugs as £31 for a goat herd with 31 animals. In transhumance herds, 95% of veterinary and drug costs is on drugs and 5% on veterinary services, so this was assumed for sedentary herds also (Majekodunmi et al., 2014). This figure was also applied to sheep, as there was no data on veterinary and drug costs for sedentary sheep in Nigeria in the literature.  This value was agreed with authors to be a reasonable average cost in sedentary herds during discussions | Non-distributed |
| SGP treatment costs/animal | 1.37 |  | Value taken from field research undertaken in Bauchi state, Nigeria. Treatment is usually antibiotics to treat secondary infections, or anti-inflammatories for pyrexia. (Limon et al., 2020) | Non-distributed |
| SGP vaccination costs/animal | 0.10 |  | There is no data on cost of SGP vaccination in Nigeria, as it is not commercially available. This is the cost of PPR vaccination in Nigeria, provided by collaborators, and is used as a proxy for SGP vaccination. This cost represents the dose and does not account for costs of delivery e.g. veterinary time, injection supplies or costs of a regional campaign e.g. social mobilisation. | Non-distributed |
| *Variable costs – marketing costs* |  |  |  |  |
| Marketing cost per animal | 0.19 |  | Costs paid for each animal sold. Half of this is for entrance to the market and half is paid for every animal sold. | Non-distributed |
|  |  |  |  |  |
| Transport costs to the market | 0.79 |  |  |  |
|  |  |  |  |  |
| Annual dues paid to the market chief/leader | 0.95 |  | Paid annually for access to the market. | Non-distributed |
| *Variable costs – labour* |  |  |  | Non-distributed |
| Labour |  |  |  |  |

^1^ Where a PERT distribution was used, the range presented is the 1^st^ and 3^rd^ quartiles, and the mean value was used in the deterministic model. Where a Uniform distribution was used, the range represents the lowest and greatest value, and the median value was used in the deterministic model.

| Supplementary Table 5. Production and economic parameters and their distributions used in stochastic simulation for transhumance herds. SGP epidemiological and vaccination parameters are presented in Table 2 of the main text. | | | | |
| --- | --- | --- | --- | --- |
|  | Value | Range^1^ | Reference/Calculation | Distribution used in stochastic simulation |
| PRODUCTION PARAMATERS |  |  |  |  |
| *Goats* |  |  |  |  |
| Offtake rate – does (proportion) | 0.302 |  | (Otte and Chilonda, 2002) | Non-distributed |
| Offtake rate – bucks (proportion) | 0.302 |  | (Otte and Chilonda, 2002) | Non-distributed |
| Mortality rate – does (proportion) | 0.15 | 0.1-0.2 | (Ameh et al., 2000; Bayer, 1983; Otte and Chilonda, 2002; Sumberg and Cassaday, 1985) | Uniform |
| Mortality rate – bucks (proportion) | 0.144 |  | (Bayer, 1983; Sumberg and Cassaday, 1985) | Non-distributed |
| Replacement rate – does (proportion) | 0.452 |  | Sum of offtake and mortality | Dependent on distributed parameters (offtake and mortality) |
| Replacement rate – bucks (proportion) | 0.446 |  | Sum of offtake and mortality | Dependent on distributed parameters (offtake and mortality) |
| Parturition rate/doe/year | 1.68 |  | (Omotayo, 2003) | Non-distributed |
| Prolificacy | 1.5 |  | (Otte and Chilonda, 2002) | Uniform |
| Mortality rate – non reproductive female goats (proportion) | 0.317 | 0.22-0.414 | (Ameh et al., 2000; Bayer, 1983; Karen Ann Dvorak, 1993; Sumberg and Cassaday, 1985) | Uniform |
| Mortality rate – non reproductive male goats (proportion) | 0.317 | 0.22-0.414 | (Ameh et al., 2000; Bayer, 1983; Karen Ann Dvorak, 1993; Sumberg and Cassaday, 1985) | Uniform |
| Herd size | 23 | 15-30 | Authors estimated based on field experience. | Uniform |
| Proportion female | 0.8 |  | Authors estimated based on field experience. | Non-distributed |
| *Sheep* |  |  |  |  |
| Offtake rate – ewes (proportion) | 0.263 |  | (Otte and Chilonda, 2002; Picardi, 2005) | Uniform |
| Offtake rate – rams (proportion) | 0.305 |  | (Picardi, 2005) | Uniform |
| Mortality rate – ewes (proportion) | 0.161 | 0.143-0.179 | (Majekodunmi et al., 2016; Otte and Chilonda, 2002) | Uniform |
| Mortality rate – rams (proportion) | 0.161 | 0.143-0.179 | (Majekodunmi et al., 2016; Otte and Chilonda, 2002) | Uniform |
| Ewe replacement rate (proportion) | 0.424 |  | Sum of offtake and mortality | Dependent on distributed parameters (offtake and mortality) |
| Ram replacement rate (proportion) | 0.466 |  | Sum of offtake and mortality | Dependent on distributed parameters (offtake and mortality) |
| Parturition rate/ewe/year | 1.09 |  | (Pablo Alarcon, manuscript in preparation) | Non-distributed |
| Prolificacy | 1.23 |  | (Ogah, 2018; Seybolt, 2015) | Uniform |
| Mortality rate – non reproductive female sheep (proportion) | 0.297 |  | (Otte and Chilonda, 2002) | Non-distributed |
| Mortality rate – non reproductive male sheep (proportion) | 0.297 |  | (Otte and Chilonda, 2002) | Non-distributed |
| Herd size | 31 | 0-68 | (Adeyinka J. Adedeji, manuscript in preparation) | Uniform |
| Proportion female | 0.8 |  | Authors experience. | Non-distributed |
| ECONOMIC PARAMATERS (£) |  |  |  |  |
| *Goats* |  |  |  |  |
| Value of healthy doe | 22.80 | 7.60-38.00 |  | Uniform |
| Value of healthy buck | 21.85 | 5.70-38.00 | Field data from focus group discussions undertaken by authors in Nigeria. | Uniform |
| Value of healthy young female goat | 19 | 9.50-28.50 | As above. | Uniform |
| Value of healthy young male goat | 11.40 | 7.60-15.20 | As above. | Uniform |
| Value of healthy doe carcass | 3.42 | 8-16 | As above. | Dependent on distributed parameter (value of animals) |
| Value of healthy buck carcass | 3.28 |  | Value of carcass is between 10-20% of value of healthy animal. 15% reduction was utilised in the deterministic model (Authors experience). | Dependent on distributed parameter (value of animals) |
| Value of healthy young female carcass | 2.85 |  | As above. | Dependent on distributed parameter (value of animals) |
| Value of healthy young male carcass | 1.71 |  | As above. | Dependent on distributed parameter (value of animals) |
| Value of SGP affected doe | 10.60 |  | As above. | Dependent on distributed parameter (value of animals) |
| Value of SGP affected buck | 10.16 |  | SGP affected goats are sold with a 44.00-60.00% reduction in price, arising from clinical manifestations of disease including weight loss, dyspnoea and skin lesions. A mean reduction of 52% was used in the deterministic model (Bolajoko et al., 2019; Limon et al., 2020) | Dependent on distributed parameter (value of animals) |
| Value of SGP affected young female goat | 8.84 |  | As above. | Dependent on distributed parameter (value of animals) |
| Value of SGP affected young male goat | 5.30 |  | As above. | Dependent on distributed parameter (value of animals) |
| Value of SGP affected doe carcass | 1.03 |  | SGP affected carcass are sold with a 70-90% reduction in value, arising from poorer carcass quality due to damage to hide and skins. A median of 80% was used in the deterministic model. (Bolajoko et al., 2019) | Dependent on distributed parameter (value of animals) |
| Value of SGP affected buck carcass | 0.98 |  | As above. | Dependent on distributed parameter (value of animals) |
| Value of SGP affected young female goat carcass | 0.85 |  | As above. | Dependent on distributed parameter (value of animals) |
| Value of SGP affected young male goat carcass | 0.51 |  | As above. | Dependent on distributed parameter (value of animals) |
| *Sheep* |  |  |  |  |
| Value of healthy ewe | 26.13 | 4.75-47.50 | Field data from focus group discussions undertaken by authors in Nigeria. | Uniform |
| Value of healthy ram | 93.10 | 34.20-152.00 | As above. | Uniform |
| Value of healthy young female sheep | 25.65 | 13.30-38.00 | As above. | Uniform |
| Value of healthy young male sheep | 35.15 | 13.30- 57.00 | As above. | Uniform |
| Value of healthy ewe carcass | 3.92 |  | Value of carcass is between 10-20% of value of healthy animal. 15% reduction was utilised in the deterministic model (Authors experience). | Dependent on distributed parameter (value of animals) |
| Value of healthy ram carcass | 13.96 |  | As above. | Dependent on distributed parameter (value of animals) |
| Value of healthy young female sheep carcass | 3.85 |  | As above. | Dependent on distributed parameter (value of animals) |
| Value of healthy young male carcass | 5.27 |  | As above. | Dependent on distributed parameter (value of animals) |
| Value of SGP affected ewe | 12.54 |  | SGP affected goats are sold with a 30.00-59.17% reduction in price, arising from clinical manifestations of disease including weight loss, dyspnoea and skin lesions A mean reduction off 44.85% was used in the deterministic model (Bolajoko et al., 2019; Limon et al., 2020) | Dependent on distributed parameter (value of animals) |
| Value of SGP affected ram | 44.69 |  | As above. | Dependent on distributed parameter (value of animals) |
| Value of SGP affected young female sheep | 12.31 |  | As above. | Dependent on distributed parameter (value of animals) |
| Value of SGP affected young male sheep | 16.87 |  | As above. | Dependent on distributed parameter (value of animals) |
| Value of SGP affected ewe carcass | 1.17 |  | SGP affected carcass are sold with a 70-90% reduction in value, arising from poorer carcass quality due to damage to hide and skins. A median of 80% was used in the deterministic model. (Bolajoko et al., 2019) | Dependent on distributed parameter (value of animals) |
| Value of SGP affected ram carcass | 4.19 |  | As above. | Dependent on distributed parameter (value of animals) |
| Value of SGP affected young female sheep carcass | 1.15 |  | As above. | Dependent on distributed parameter (value of animals) |
| Value of SGP affected young male sheep carcass | 1.58 |  | As above. | Dependent on distributed parameter (value of animals) |
| *Veterinary and medicine costs* |  |  |  |  |
| Drug costs/animal | 1.28 |  | A Nigerian study found the total annual cost of veterinary and drugs as £264.70 for 188 animals, with 95% of this cost on drugs and 5% on veterinary services (Majekodunmi et al., 2017). This study only considered cattle, however authors in Nigeria agreed that this is a reasonable average costs of transhumance expenditure on small ruminant veterinary services and drugs | Non-distributed |
| Veterinary costs/  animal | 0.05 |  | As above. | Non-distributed |
| SGP treatment costs/animal | 1.84 |  | Value taken from field research undertaken in Bauchi state, Nigeria. Treatment is usually antibiotics to treat secondary infections, or anti-inflammatories for pyrexia. (Limon et al., 2020) | Non-distributed |
| SGP vaccination costs/animal | 0.10 |  | There is no data on cost of SGP vaccination in Nigeria, as it is not commercially available. This is the cost of PPR vaccination in Nigeria, provided by collaborators, and is used as a proxy for SGP vaccination. This cost represents the dose and does not account for costs of delivery e.g. veterinary time, injection supplies or costs of a regional campaign e.g. social mobilisation. | Non-distributed |
|  |  |  |  |  |
|  |  |  |  |  |
| *Variable costs – marketing costs* |  |  |  |  |
| Marketing cost per animal | 0.19 |  | Costs paid for each animal sold. Half of this is for entrance to the market and half is paid for every animal sold. Authors experience in field and focus group discussions with farmers. | Non-distributed |
|  |  |  |  |  |
| Transport costs to the market | 0.79 |  | Cost of taking animals to market. Authors experience in field and focus group discussions with farmers. | Non-distributed |
|  |  |  |  |  |
| Annual dues paid to the market chief/leader | 0.95 |  | Paid annually for access to the market. Authors experience in field and focus group discussions with farmers. | Non-distributed |
|  |  |  |  |  |
|  |  |  |  |  |
|  |  |  |  |  |
|  |  |  |  |  |
| *Variable costs – labour* |  |  |  |  |
| Labour | 123.50 |  | Extra labour in transhumance herds is in the form of herders. They are either given a male cow (approximately 2 years old), or cash. Use an average of one herder per year. Obtained from focus group discussions with farmers by authors. |  |

^1^ Where a PERT distribution was used, the range presented is the 1^st^ and 3^rd^ quartiles, and the mean value was used in the deterministic model. Where a Uniform distribution was used, the range represents the lowest and greatest value, and the median value was used in the deterministic model.

|  | | |  |
| --- | --- | --- | --- |
| **Supplementary Table 6.** Economic values and cost structure utilised for regional vaccination programme. All costs were obtained from authors experience in the field, using costs of PPR vaccination delivery as a proxy for cost of SGP vaccination programmes. | | | |
|  | **Costs (£)** | **Quantity required per local government area** | **Justification** |
| ***Consumables*** |  |  |  |
| Cold Box | 57.00 | 2 | Required transportation of SGP vaccination. |
| Alcohol (2.5) L | 15.20 | 2 | Required for delivery of vaccination. |
| Cotton wool (absorbent) | 3.80 | 3 | As above. |
| Hand gloves | 6.65 | 10 | As above. |
| Disposable lab coats | 6.65 | 36 | Personal protection equipment required for staff. |
| Disinfectant | 38.00 | 1 | Required for delivery of vaccination. |
| Detergent (1 litre) | 0.95 | 5 | As above. |
| Trash/sharps cans | 4.75 | 5 | Required for safe disposal of syringes and needles. |
| Syringes/needles | 28.50 | 2 | As above. |
| First aid | 16.15 | 1 | Required for staff safety. |
| Ice packs (gel) | 1.90 | 20 | Required transportation of SGP vaccination |
| Hand soap (500ml) | 0.95 | 5 | Required for hygiene of staff. |
| Hand sanitiser (5000 ml) | 2.85 | 10 | Required for hygiene of staff. |
| Boots | 4.75 | 5 | Personal protection equipment required for staff. |
| Stationary | 19.00 | 1 | Required for record keeping during vaccination programme. |
| Face mask | 5.70 | 2 | Personal protection equipment required for staff. |
| Storage box | 47.50 | 2 |  |
| Fuel | 11.40 | 15 with SGP only, 5 with SGP and PPR | Required transportation of staff. |
| ***Staff*** |  |  |  |
| **Veterinarian** | 47.50 | 15 with SGP only, 5 with SGP and PPR | One veterinarian required. |
| Vaccinators | 22.80 | 15 with SGP only, 5 with SGP and PPR | Two vaccinators required. |
| Driver | 22.80 | 15 with SGP only, 5 with SGP and PPR | One driver required. |
| ***Sensitisation*** |  |  |  |
| LGA staff | 68.40 |  | Costs for three staff. |
| Posters | 190.00 |  | Required to increase farmer engagement with vaccination. |

**References**

Adu, I.F., Buvanendran, V., Lakpini, C.A.M., 1979. The Reproductive Performance Of Red Sokoto Goats In Nigeria. J. Agric. Sci. 93, 563–566. https://doi.org/10.1017/S002185960003896X

Ajala, MK, Lamidi, OS, Otaru, S., 2008. Peri-Urban Small Ruminant Production in Northern Guinea Savanna, Nigeria. Asian J. Anim. Vet. Adv.

Ajala, M.K., Lamidi, O.S., Otaru, S.M., 2008. Peri-Urban Small Ruminant Production in Northern Guinea Savanna, Nigeria. Asian J. Anim. Vet. Adv. 3, 138–146. https://doi.org/10.3923/ajava.2008.138.146

Ameh, J.A., Egwu, G.O., Tijjani, A.N., 2000. Mortality in sahelian goats in Nigeria. Prev. Vet. Med. 44, 107–111. https://doi.org/10.1016/S0167-5877(99)00108-7

Bayer, W., 1983. Paper 7 : Traditional small ruminant production in the subhumid zone of Nigeria.

Bolajoko, M.B., Adedeji, A.J., Dashe, G.D., Òsemeke, O.H., Luka, P.D., 2019. Molecular Epidemiology and Economic Impact of Goat Pox on Small Holder Sheep and Goats Farmers in North Central Nigeria. Small Rumin. Res. 179, 75–78. https://doi.org/10.1016/j.smallrumres.2019.09.013

Karen Ann Dvorak, 1993. Social Science Researchfor Agricultural Technology Development.

Limon, G., Gamawa, A.A., Ahmed, A.I., Lyons, N.A., Beard, P.M., 2020. Epidemiological Characteristics and Economic Impact of Lumpy Skin Disease, Sheeppox and Goatpox Among Subsistence Farmers in Northeast Nigeria. Front. Vet. Sci. 7, 1–13. https://doi.org/10.3389/fvets.2020.00008

Majekodunmi, A.O., Dongkum, C., Langs, T., Shaw, A., Welburn, S., 2016. Improved productivity and sustainable pastoral systems in an era of insecurity—Fulani herds of the southern Jos Plateau, North-Central Nigeria. Trop. Anim. Health Prod. 48, 1719–1728. https://doi.org/10.1007/s11250-016-1149-8

Majekodunmi, A.O., Dongkum, C., Langs, T., Shaw, A.P.M., Welburn, S.C., 2017. Shifting livelihood strategies in northern Nigeria - extensified production and livelihood diversification amongst Fulani pastoralists. Pastoralism 7. https://doi.org/10.1186/s13570-017-0091-3

Majekodunmi, A.O., Fajinmi, A., Dongkum, C., Shaw, A.P.M., Welburn, S.C., 2014. Pastoral livelihoods of the Fulani on the Jos Plateau of Nigeria. Pastoralism 4, 1–16. https://doi.org/10.1186/s13570-014-0020-7

Ogah, D.M., 2018. PRODUCTIVITY MEASURES AND CHALLENGES FACING SMALL HOLDER LIVESTOCK FARMERS IN HUMID NORTH CENTRAL NIGERIA.

Omotayo, A.M., 2003. Ecological implications of fulbe pastoralism in southwestern Nigeria. L. Degrad. Dev. 14, 445–457. https://doi.org/10.1002/ldr.565

Otte, M.J., Chilonda, P., 2002. Cattle and Small Ruminant Production Systems in Sub Aharan. Fao 1–98.

Picardi, A., 2005. A systems analysis of pastoralism in the West African Sahel.

Seybolt, T., 2015. Governance and Development in Africa 315–324. https://doi.org/10.1201/b18455-24

Sumberg, J., Cassaday, K., 1985. Sheep and goats in humid West Africa. Small Rumin. Prod. Syst. humid Zo. West Africa 23–26.
